# Supplementary material for: Activation of the IRE1 RNase through remodeling of the kinase front pocket by ATP-competitive ligands
Source: Nat Commun. 2020 Dec 14;11:6387. doi: 10.1038/s41467-020-19974-5 (PMC7736581; doi:10.1038/s41467-020-19974-5)
Supplement: Supplementary file 1 — Supplementary Information [file 41467_2020_19974_MOESM1_ESM.pdf]

Supplementary Information

Activation of the IRE1 RNase Through Remodeling of the Kinase Front Pocket by ATP-competitive ligands

Elena Ferri[a,b], Adrien Le Thomas[c], Heidi Ackerly Wallweber[a], Eric S. Day[d], Benjamin T. Walters[e], Susan E. Kaufman[e], Marie-Gabrielle Braun[b], Kevin R. Clark[e], Maureen H. Beresini[e], Kyle Mortara[f], Yung-Chia A. Chen[c], Breanna Canter[b], Wilson Phung,[g] Peter S. Liu,[g] Alfred Lammens[h], Avi Ashkenazi[c], Joachim Rudolph[b]* (rudolph.joachim@gene.com), Weiru Wang[a]*(wang.weiru@gene.com).

Genentech, Inc., 1 DNA way, South San Francisco, CA 94080.

[a]Structural Biology; [b]Discovery Chemistry; [c]Cancer Immunology; [d]Pharmaceutical Development; [e]Biochemical and Cellular Pharmacology; [f]BioMolecular Resources; [g] Microchemistry, Proteomics & Lipidomics.

[h]*Proteros biostructures GmbH, Bunsenstr. 7a, D - 82152 Martinsried, Germany*

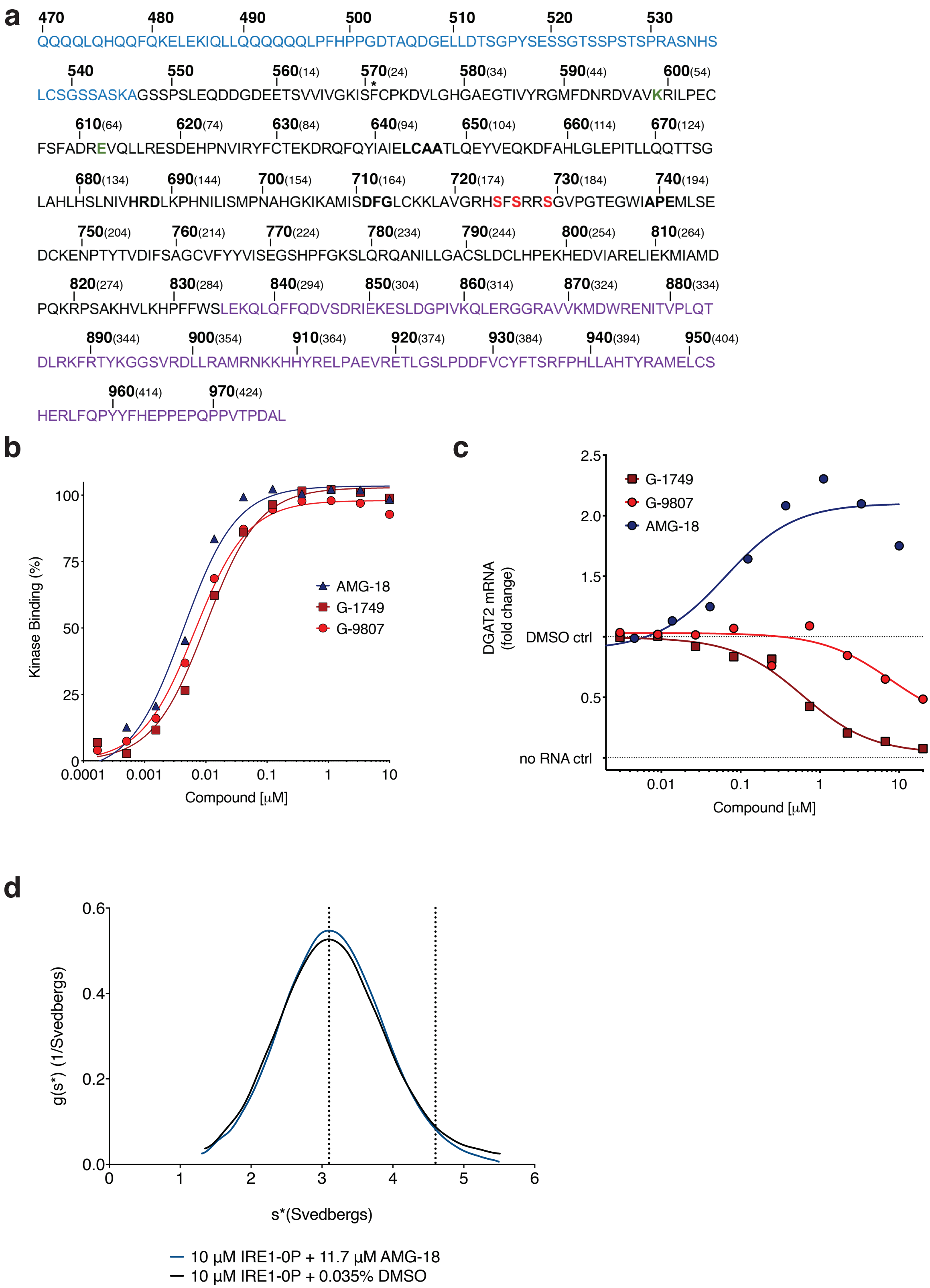

Supplementary Figure 1. Characterization of IRE1 kinase inhibitors.
a, Sequence of the human IRE1 constructs used in this work. The IRE1 KR construct (G547-L977) is shown in black and purple. The kinase domain (black) starts at F571(*), as annotated in the Uniprot

website. The RNase domain is shown in purple. The linker region included in the IRE1 LKR construct (Q470-L977) is shown in blue. The activation and αEF-αF loops are also indicated, with the phosphorylation sites S724, S726, S729 reported in bold red. Key residues such as the hinge region (L644-A647), the HRD, DFG, and APE motifs are shown in bold black. The salt bridge K599-E612 is shown in bold green. Sequence numbering as reported in the HX-MS multiplots is also indicated in parenthesis. **b**, IRE1 kinase binding of compounds in Figure 1a measured by competitive ATP-site binding TR-FRET assay. Source data are provided as a Source Data file. Data are presented as the mean of measurements from two independent experiments (n = 2). **c**, RNA levels for IRE1 RNase RIDD substrate DGAT2 measured by bDNA assay of lysates from KMS-11 cells treated with compounds for four hours. Background from the no RNA control was subtracted from signal before calculating fold change. Source data are provided as a Source Data file. Data are presented as the mean of measurements from two independent experiments (n = 2). **d**, SV-AUC experiments for IRE1 LKR (Q470-L977) unphosphorylated in the presence of slight excess AMG-18. Calculated sedimentation coefficients for IRE1 monomer (3.1 svedbergs) and dimer (4.6 svedbergs) are highlighted by dotted lines. Source data are provided as a Source Data file.

a

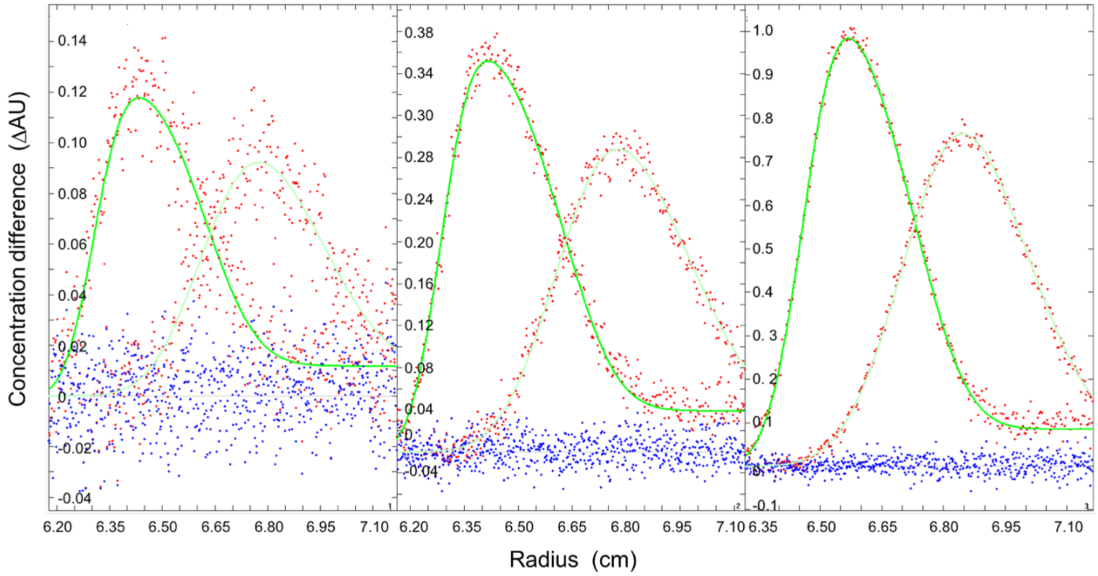

MW (monomer) = 56.3 kDa

S (monomer) = 3.19 s
S (dimer) = 4.55 s
std dev = 0.015

K_d = 67 μM

b

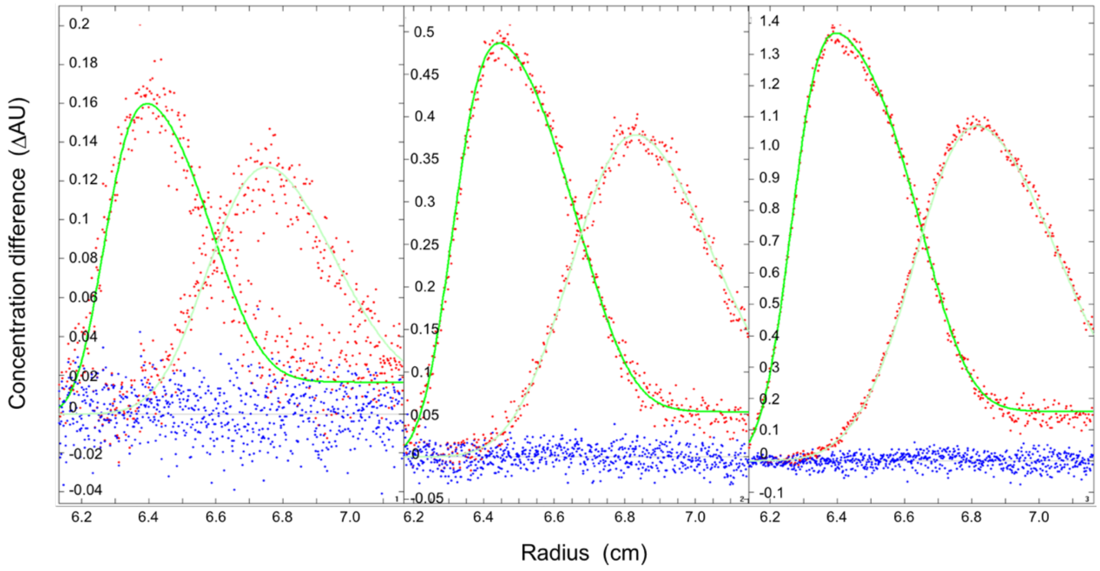

Mw (Monomer) = 57.2 kDa

S(Monomer) = 3.18 s
S(Dimer) = 4.78 s

K_d = 35 μM

Std dev = 0.015

Supplementary Figure 2. Fit profile of SV-AUC sedimentation velocity experiments to a monomer-dimer equilibrium model (A <- -> A_2) from Sedanal software.
a, IRE1 LKR auto-phosphorylated; **b**,IRE1 LKR unphosphorylated + G-9807.

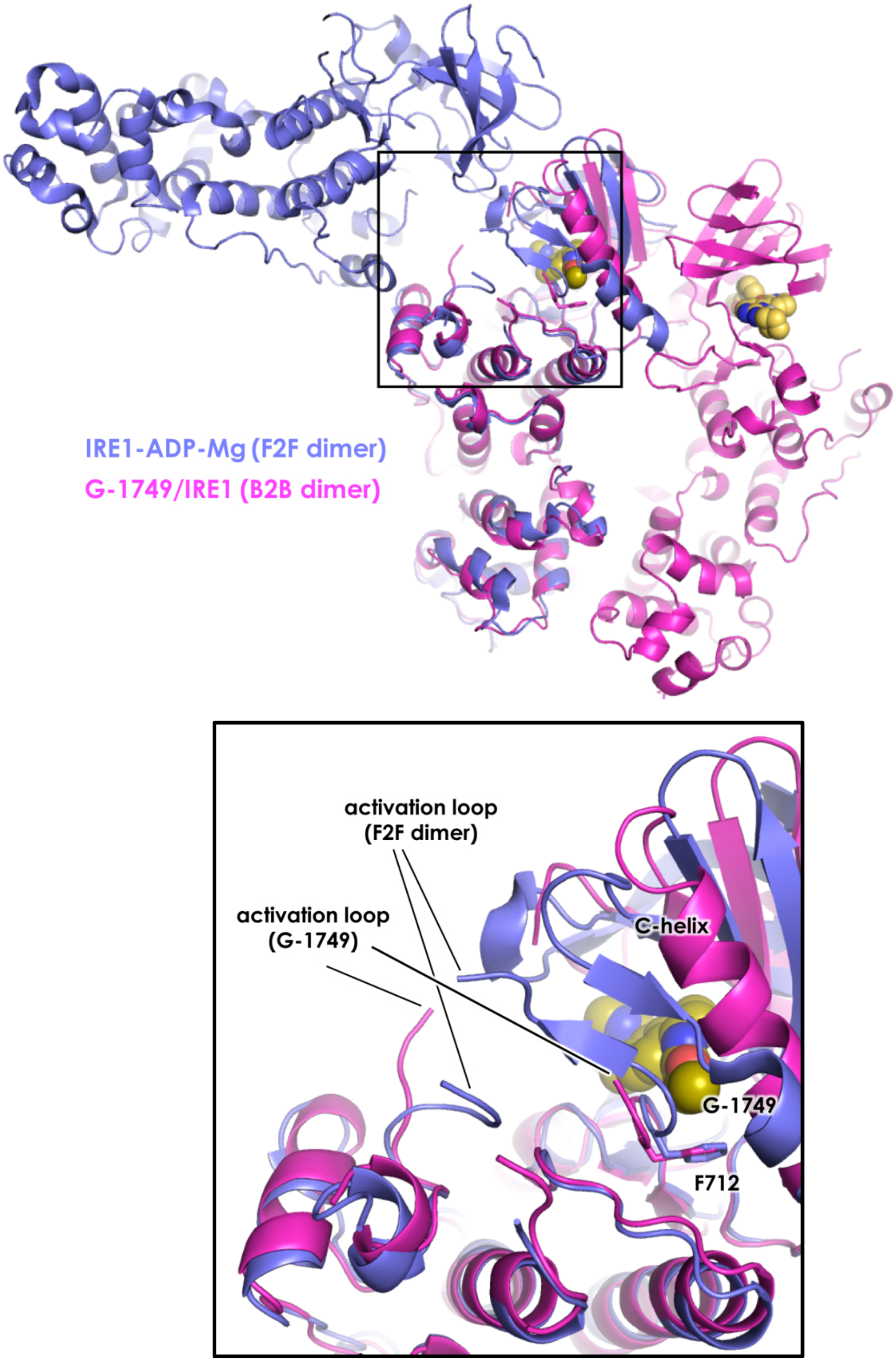

Supplementary Figure 3. Comparison between G-1749/IRE1 back-to-back dimer and IRE1/ADP face-to-face dimer.
Top: overlay of IRE1-ADP-Mg crystal structure (PDB: 3P23) of face-to-face dimer (F2F) in blue and IRE1-G-1749 crystal structure (back-to-back, B2B, dimer) in magenta.
Bottom, inset: detail of activation loop and residue F712 in the two structures. G-1749 is shown as gold spheres.

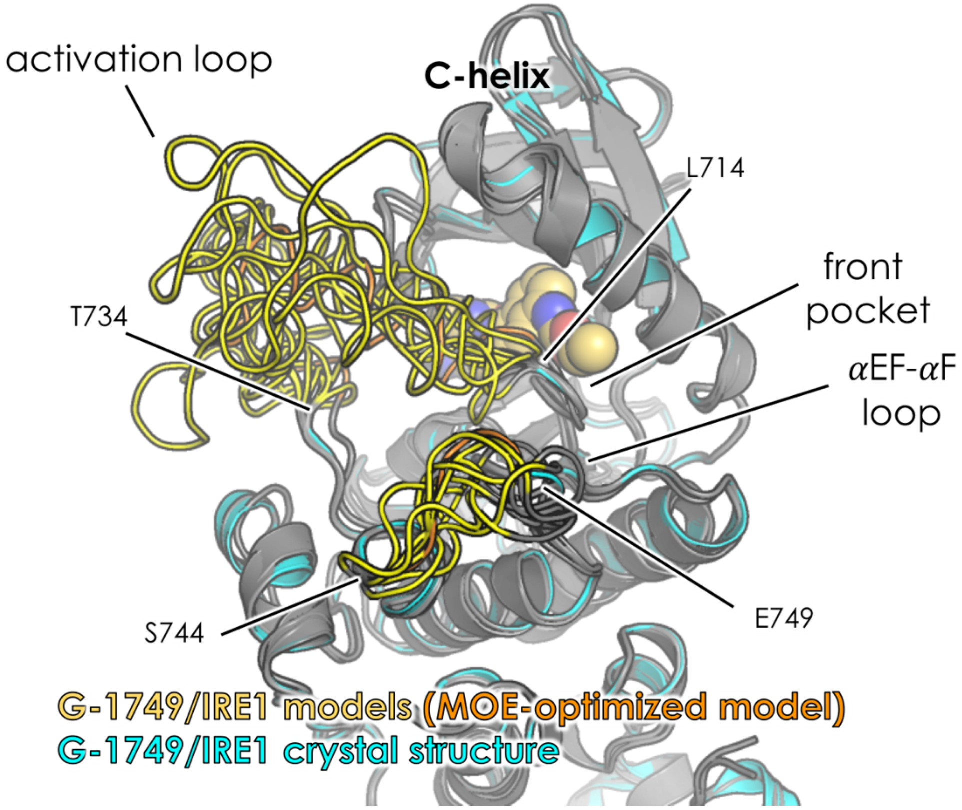

Supplementary Figure 4. Modelling of the IRE1-G-1749 complex loops for which density is absent in crystallographic data.
Ten lowest energy conformation models of the IRE1/G-1749 complex as calculated by the Homology Model algorithm in the Molecular Operating Environment software (MOE) using IRE1/G-1749 complex structure (chain A) as template. The crystal structure is shown in cyan. The modeled loop segments that are missing in the crystallographic data are highlighted in yellow. The remaining protein chain is shown in dark gray. The orange model is the one reported in Figure 4a.

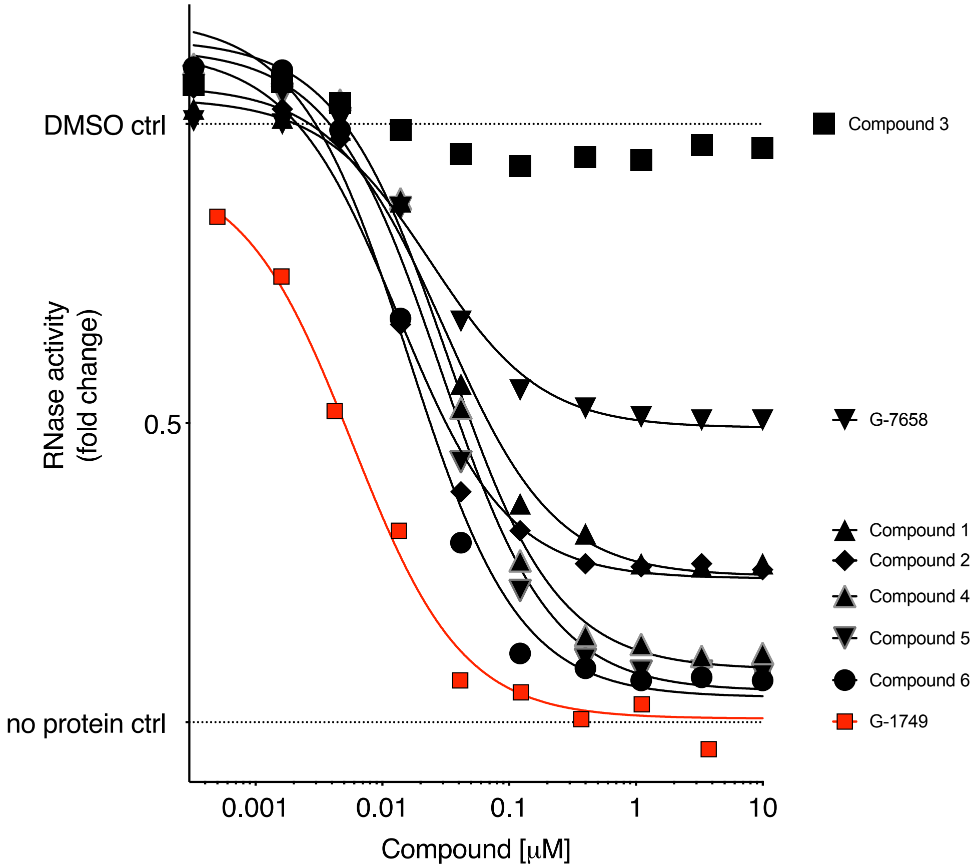

Supplementary Figure 5. RNA cleavage assay with IRE1 KR 3P in the presence of G-1749 and derivative compounds shown in Figure 3.
IRE1 RNase activity was measured by a kinetic fluorescence assay (see Methods for detailed substrate and assay description). Background from the no-protein control was subtracted from signal before calculating fold change. Details of the assay are in the Methods. Source data are provided as a Source Data file. Data are presented as the mean for measurements from two independent experiments (n = 2).

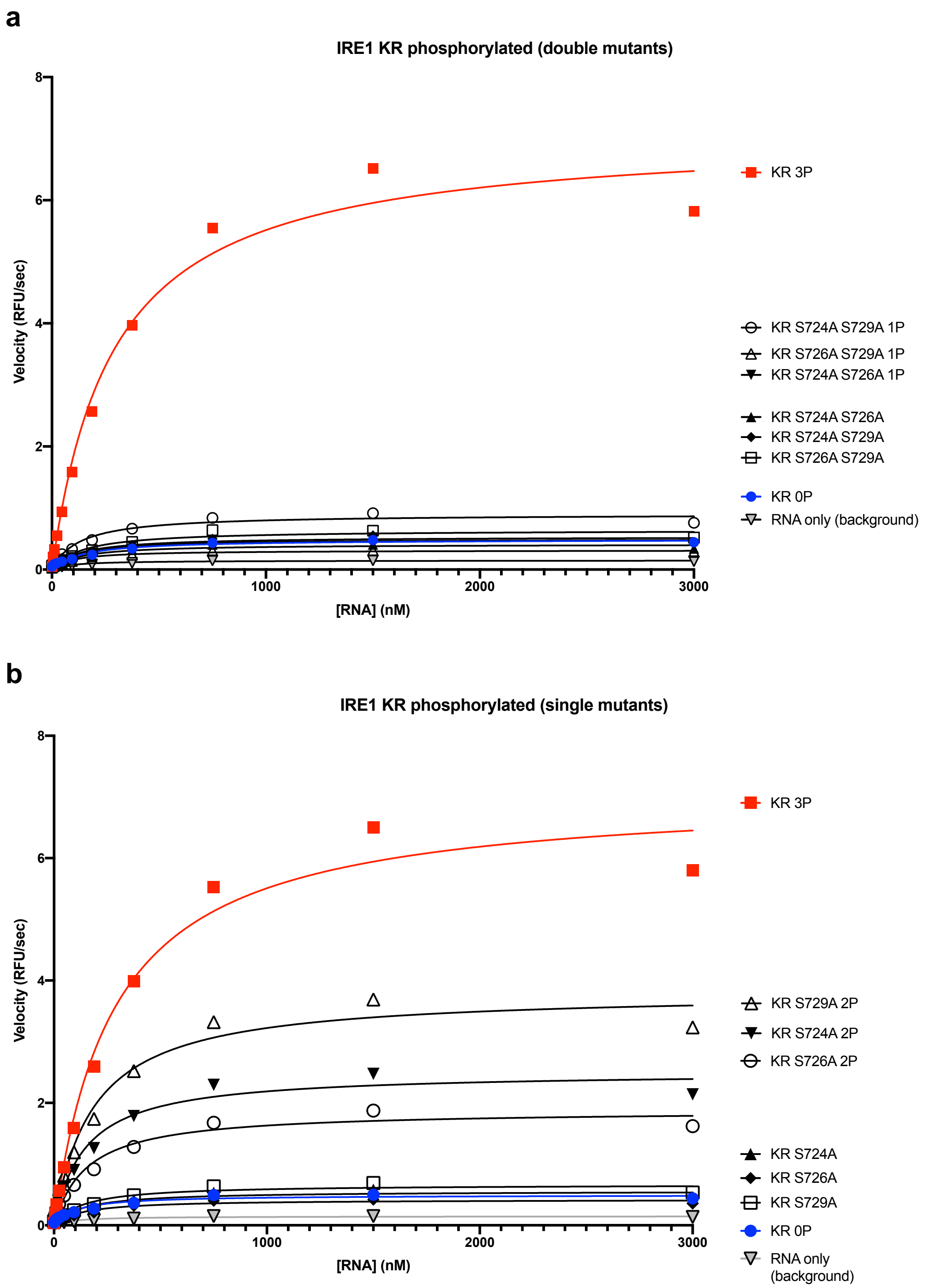

Supplementary Figure 6. Effect of single and double phosphorylation on the activity of IRE1 KR RNase measured in an RNA cleavage assay.
The velocity of IRE1 KR RNase is reported as Relative Fluorescence Units (RFU)/second as a function of the concentration of the RNA substrate (see Methods for detailed substrate and assay description) **a**, Double mutants of activation loop serine residues, singly phosphorylated protein; **b**, Single mutants of activation loop serine residues, doubly phosphorylated protein. Source data are provided as a Source Data file. Data are presented as the mean for measurements from two independent experiments (n = 2).

a

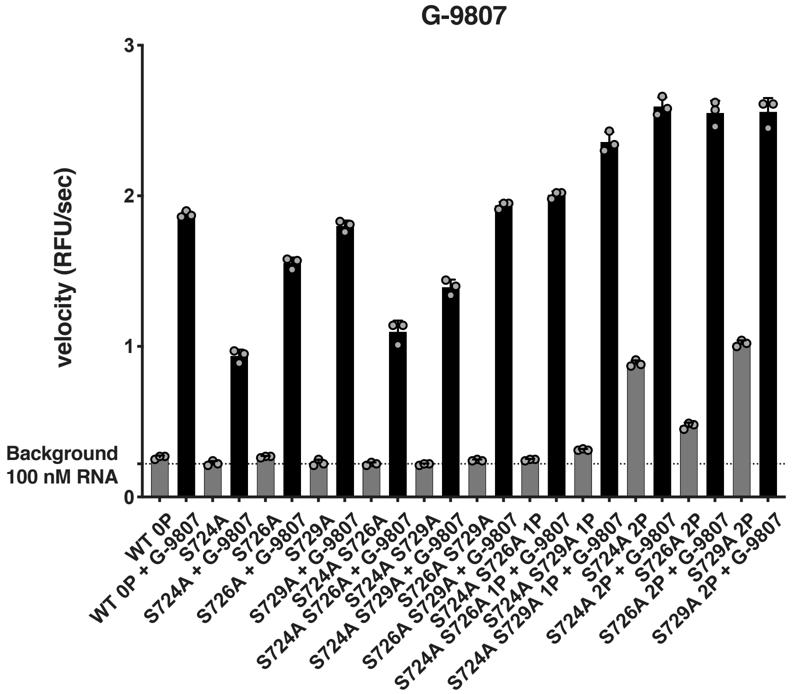

b

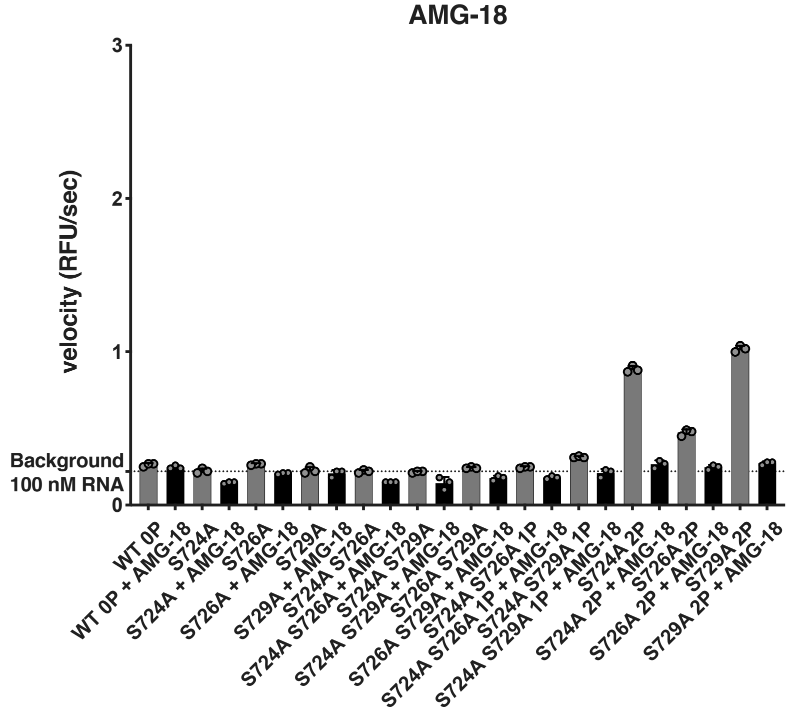

Supplementary Figure 7. RNA cleavage assays with S/A mutants of IRE1 KR in the presence of G-9807 (a) and AMG-18 (b).

Protein concentration 8 nM, RNA 100 nM, compounds 10 μM. The velocity of IRE1 KR RNase is reported as Relative Fluorescence Units (RFU)/second as a function of the concentration of the RNA substrate (see Methods for detailed substrate and assay description). Source data are provided as a Source Data file. Data are presented as the mean and S.D. for measurements from three independent experiments (n = 3).

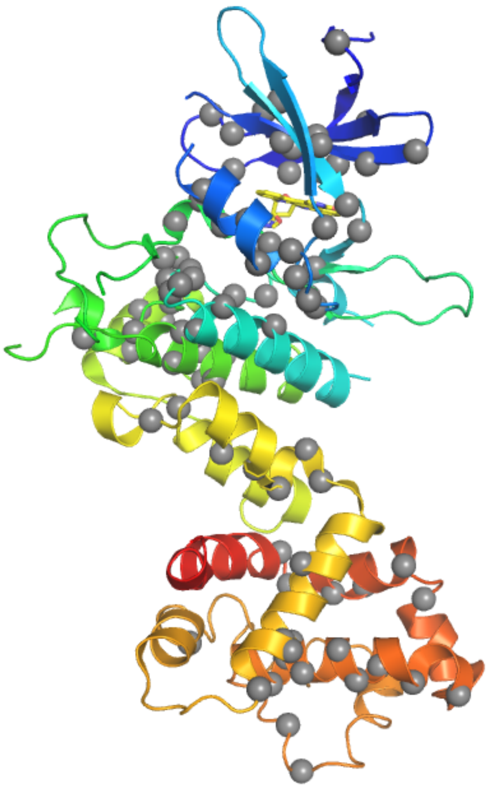

Supplementary Figure 8. **Structural map of 100% evolutionarily conserved residues from yeast to human.**
The monomer of IRE1 is shown as cartoon, colored as spectrum from blue (N-terminus of the kinase domain) to red (C-terminus of the RNase domain). The Cα atoms of the conserved residues are shown as gray spheres. The conserved residues were identified using the Consurf server (https://consurf.tau.ac.il/) using Chain A of the crystal structure of IRE1 in complex with Staurosporine (shown as yellow stick) (PDB: 4YZC) as input. The alignment calculation was run on 150 sequences that represent the 347 unique homologues identified by the Consurf algorithm among 875 homologs in the UniProt database.

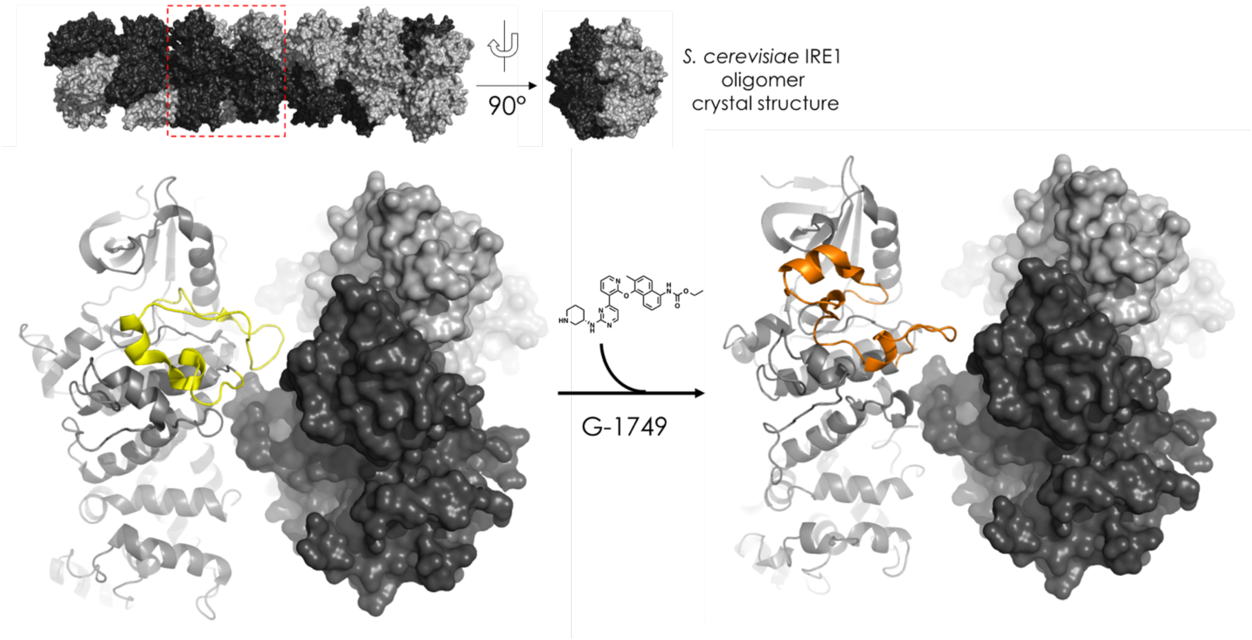

Supplementary Figure 9 – G-1749 may affect the oligomeric structure of IRE1.
S. cerevisiae IRE1 oligomeric structure (PDB: 3SDJ) is shown as surface in the upper panel. Each back-to-back (B2B) dimer is composed of a black monomer and a gray monomer. The oligomer's tetrameric unit is highlighted in a red box and shown in the lower panel with one dimer as cartoon and one dimer as surface. The activation and αEF-αF loops are highlighted in yellow (yeast, apo) or orange (human, upon binding of Activator G-1749).

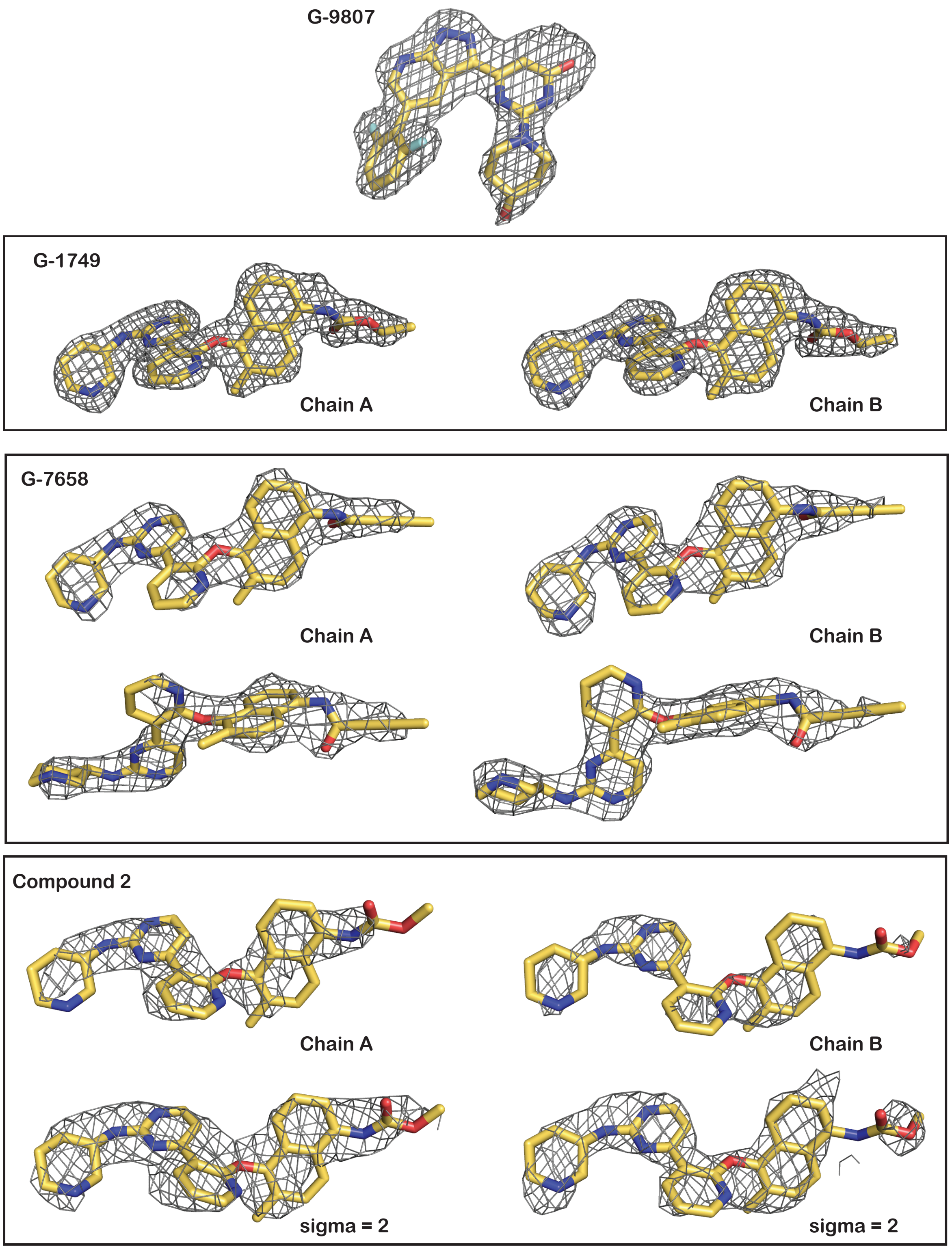

Supplementary Figure 10. Fo-Fc omit maps for all compounds co-crystallized with IRE1.
The maps are visualized at contour level sigma=3, unless specified, and within 2 Å of the compound.

a.

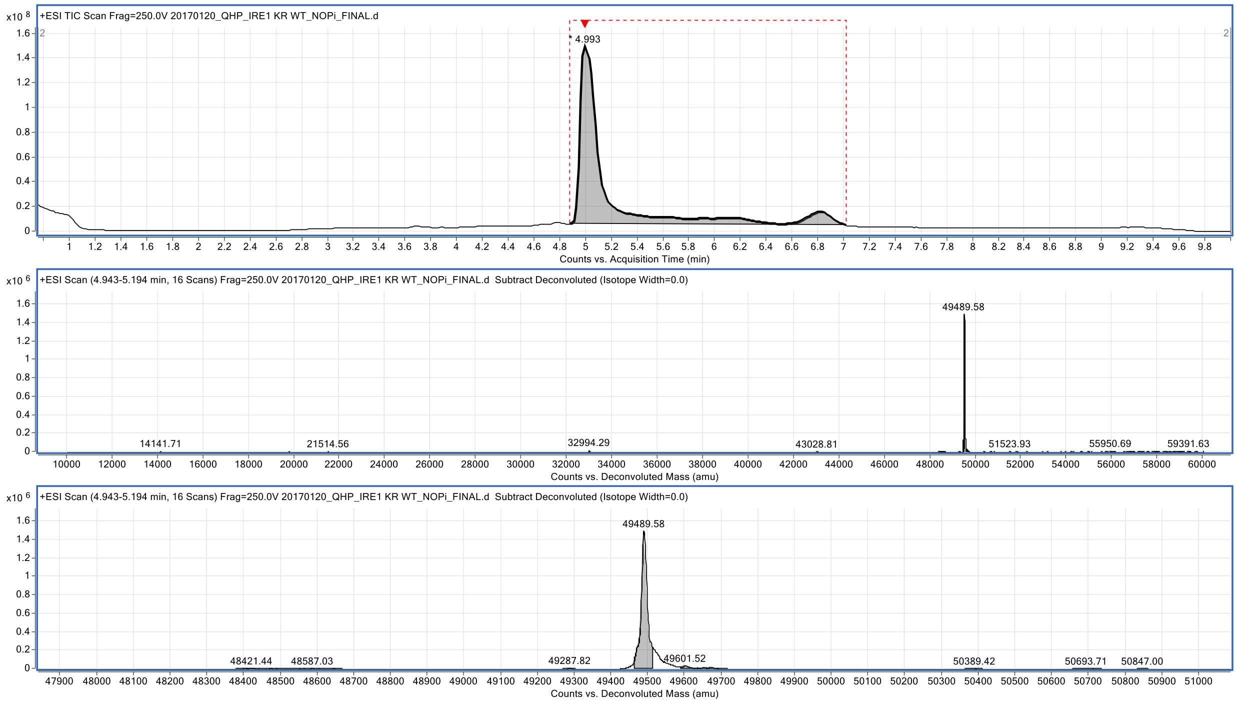

b.

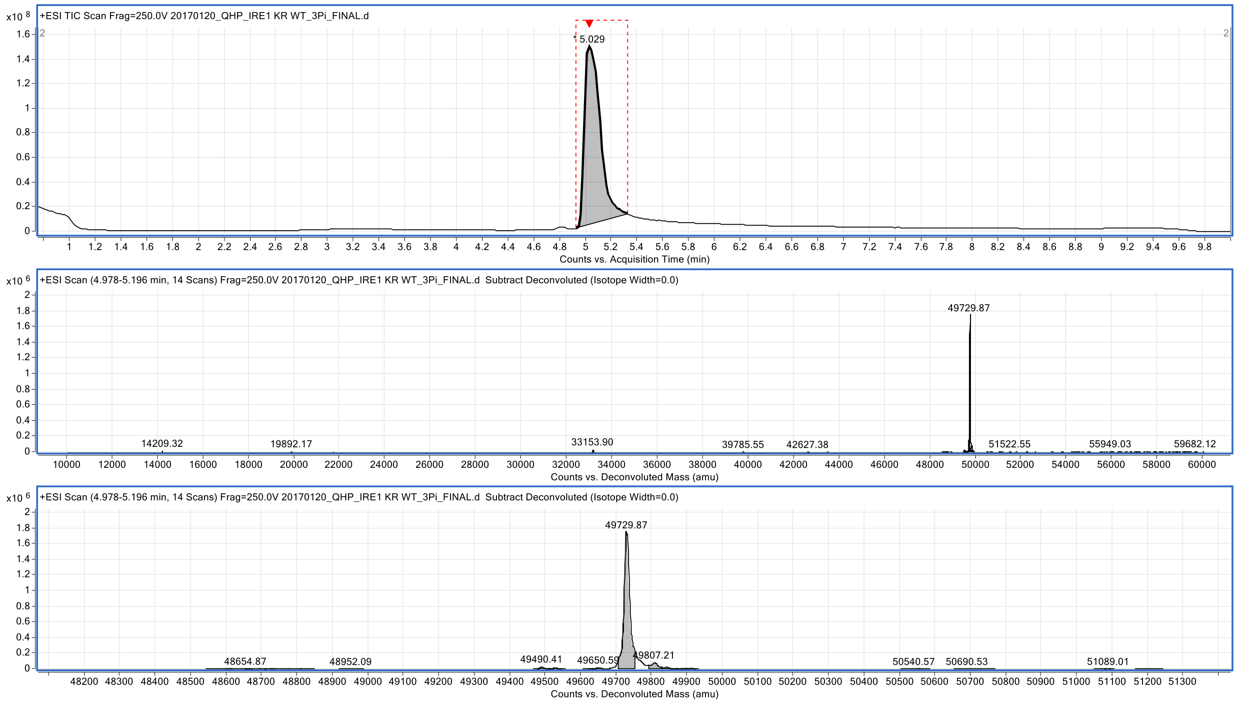

Supplementary Figure 11. Representative LC-MS chromatograms for IRE1 protein purity and identity.

Proteins were injected onto a reverse-phase HPLC analytical column and eluted using a gradient from 100% water to 40%water-60% acetonitrile over six minutes. **a**, IRE1 0P (G547-L977) – Expected MW = 49489 Da. Found: 49489.58 Da. **b**, IRE1 3P (G547-L977) – Expected MW = 49729 Da. Found: 49729.87 Da.

Supplementary Table 1.
PERK inhibition by Reaction Biology (^{33}P incorporation assay with 10 mM myelin basic protein as substrate, 10 mM ATP and compound at 1 mM).

Compound	Replicate 1	Replicate 2	Mean % Inhibition
**AMG-18**	4.3	6.5	5.4
**G-9807**	1.5	7.5	4.5
**G-1749**	1	18.8	9.9

Supplementary Table 2.
Assay values and respective standard deviations from graphs in Figure 1 and 3.

**Compound**	**EC_{50} (RNase biochemical assay, μM)**	**RNase modulation (RNase biochemical assay, fold change)**	**EC_{50} (KMS-11 bDNA assay, XBP1s mRNA μM)**	**XBP1s mRNA levels (KMS-11 bDNA assay, fold change)**
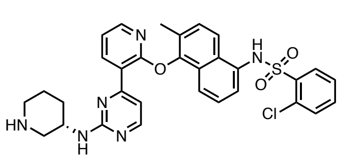 **AMG-18**	0.017±0.004	-0.021±0.013	0.193±0.036	-0.077±0.038
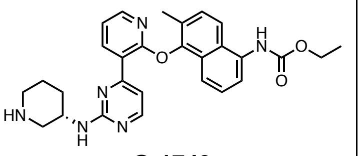 **G-1749**	0.009±0.003	1.864±0.056	1.310±0.168	8.255±0.222
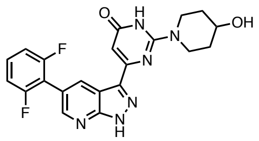 **G-9807**	0.018±0.003	3.504±0.056	1.769±0.691	7.213±0.587
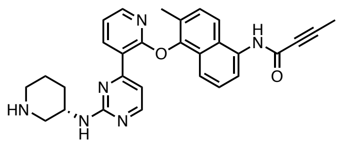 **G-7658**	0.007±0.001	2.674±0.038	0.223±0.033	10.200±0.240
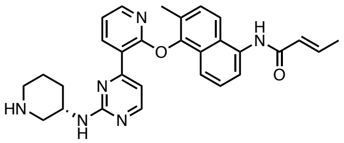 **1**	0.007±0.001	2.381±0.028	0.331±0.128	8.564±0.551

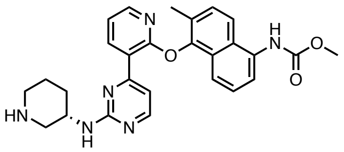 **2**	0.004±0.004	1.389±0.056	3.373±0.709	4.842±0.254
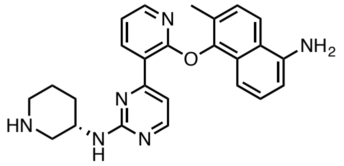 **3**	0.031±0.023	1.300±0.024	3.262±0.480	7.543±0.296
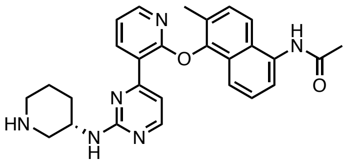 **4**	0.008±0.002	0.334±0.025	0.649±0.241	0.239±0.067
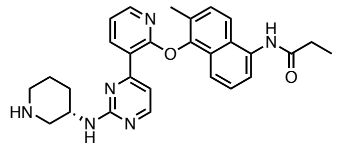 **5**	0.028±0.005	0.272±0.015	0.816±0.346	0.282±0.070
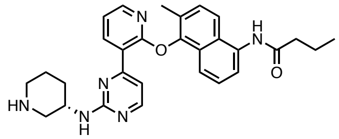 **6**	0.022±0.035	0.226±0.013	0.221±0.052	0.197±0.034
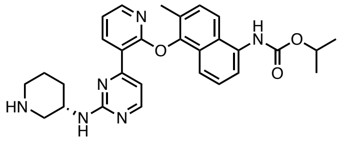 **7**	0.036±0.005	0.135±0.017	0.456±0.226	-0.171±0.107
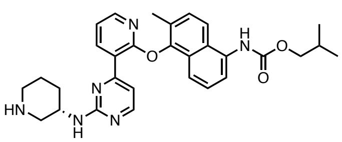 **8**	0.018±0.003	0.043±0.022	0.465±0.154	-0.347±0.074
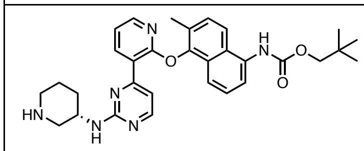 **9**	0.068±0.021	0.031±0.043	1.049±0.372	-0.368±0.092
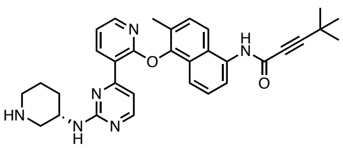 **10**	0.035±0.005	-0.022±0.019	0.380±0.109	-0.207±0.067

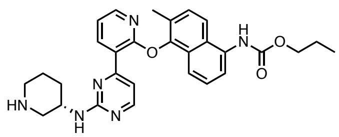 **11**	0.029±0.006	0.196±0.021	0.234±0.078	-0.095±0.059
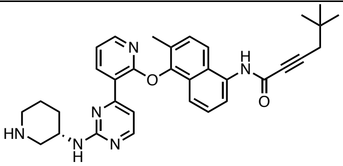 **12**	0.052±0.012	0.025±0.028	0.645±0.111	-0.227±0.045

Supplementary Table 3
Phosphorylation site mapping for IRE1 KR WT 3P, IRE1 S/A and X/A activation loop mutants, and IRE1 LKR autophosphorylated, performed by LC-MS/MS analysis following protease digestion. Yes* denotes observed S726 phosphorylation for which quantification was not possible due to the presence of a miscleaved peptide containing phosphorylated S726.

**Protein**	**% phosphorylation**						
	**S548/S549**	**S551**	**T561/S562**	**S724**	**S726**	**S729**	**T973**
KR WT 3P	N/A	N/A	N/A	**82**	**Yes***	**52**	N/A
**Mutant (IRE1 KR)**	**% phosphorylation**						
	**S548/S549**	**S551**	**T561/S562**	**S724**	**S726**	**S729**	**T973**
S724A	N/A	N/A	N/A	**-**	**>90**	**>90**	low
S726A	N/A	N/A	N/A	**90**	**-**	**71**	low
S729A	N/A	N/A	N/A	**82**	**Yes***	**-**	N/A
S724A S726A	N/A	N/A	N/A	**-**	**-**	**97**	N/A
S726A S729A	N/A	N/A	N/A	**89**	**-**	**-**	N/A
S724A S729A	N/A	N/A	N/A	**-**	**Yes***	**-**	N/A
**Mutant (IRE1 KR)**	**% phosphorylation**						
	**S548/S549**	**S551**	**T561/S562**	**S724**	**S726**	**S729**	**T973**
R611A	2.4	1.3	4.3	**72.4**	**Yes***	**78.2**	9.3
K716A	<1	<1	1.2	**79.1**	**Yes***	**60.3**	3.8
R687A	2.8	<1	3.7	**88**	**Yes***	**91.1**	8.6
R611A, K716A	1.1	<1	<1	**99.4**	**Yes***	**92.1**	4.7

R611A. R687A	13	10	5	**99.9**	**Yes***	**89.3**	21.2
R687A, K716A	11.6	9.2	3.2	**95.4**	**Yes***	**98.1**	26.2
R611A, R687A, K716A	5.5	1	4.2	**95.3**	**Yes***	**96.1**	19.9
R722A	4.5	1.1	5	**68.3**	**48.1**	**91.5**	11.6
N750A	<1	<1	1.3	**89.3**	**Yes***	**94**	14
R722A, N750A	1.5	<1	1.8	**76.7**	**26.6**	**90.6**	5.4
R611A, R687A, K716A, R722A, N750A	4.9	1.6	4.6	**89.8**	**52.3**	**91.8**	13.3

**Protein**	**% phosphorylation**						
	**S514**	**S533**	**S724**	**S726**	**S729**	**S834**	**T891**
IRE1 LKR WT (autophosphorylated)	low	42	**79**	**20**	**51**	<5	low

Supplementary Table 4.
Sequences of oligonucleotides utilized in this study.

Name	Sequence
RNase assay substrate	5′FAM-CAUGUCCGCAGCGCAUG-3′BHQ<br>5′-Carboxyfluorescein (FAM)<br>3′-Black Hole Quencher (BHQ)
IRE1 CRISPR KO gRNA pair	CTTGTTGTTTGTGTCAACGC<br>TCTTGCTTCCAAGCGTATAC
bDNA XBP1s capture probes<br><br>SA-50209-01 probe set in QuantiGene kit by Thermofisher	AGTTTTTGGTTCTCTTCTTCTAAATCTA<br>TCGTAAAAGCTGATTTTCTAGCAA<br>CCGCCTCCTCTTCAGCAA<br>TCACTTCATTCCCCTTGGCTT<br>GGGTCCAAGTTGTCCAGAATG<br>AGGGCATTTGAAGAACATGACT
bDNA DGAT2 capture probes<br><br>SA-10160 probe set in QuantiGene kit by Thermofisher	CGATGATGATAGCATTGCCACT<br>GCAGGGCCAGTTTCACAAAG<br>GCCCCAGGAGCCCTCCT<br>GGAACTTCTTCTGGACCCATCG<br>CAGGTGTCGGAGGAGAAGAGG<br>TGGGTGCTCCAGCTTGGG

Supplementary Methods

Compound synthesis and characterization

G-9807 was synthesized according to Supplementary Figure 12.

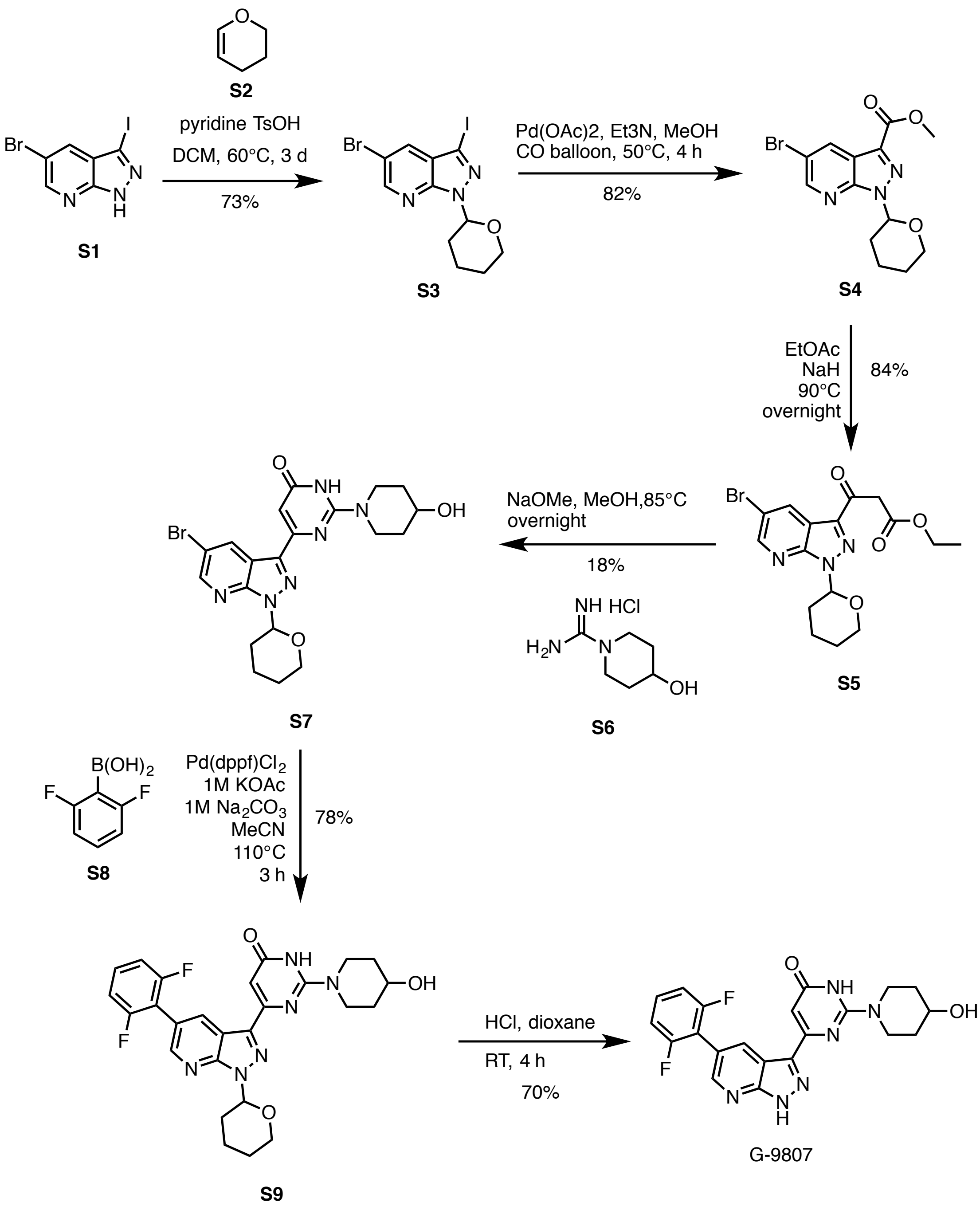

Supplementary Figure 12 – Synthesis of G-9807.
The synthesis of G-9807 from commercial reagents is reported. Yields are reported for each step.

Synthesis of G-9807 intermediate **S3**:

To a solution of compound **S1** (7.50 g, 23.1 mmol) in DCM (80 mL) was added **S2** (4.20 g, 50.0 mmol) and pyridine·TsOH (1.20 g, 6.32 mmol). The mixture was stirred at 60 ºC overnight. It was then diluted with DCM (200 mL), washed with $NaHCO_3$ (200 mL), 5% citric acid aq. solution (200 mL), and brine (200 mL), dried over sodium sulfate, and concentrated under vacuum. The residue was purified by column chromatography using 10% EtOAc in hexanes as eluents to give compound **S3** (6.89 g, 16.9 mmol, 73%) as a white solid.

Synthesis of G-9807 intermediate **S4**:

To a solution of compound **S3** (6.89 g, 16.9 mmol) in MeOH (120 mL) was added Et_3N (5 mL) and $Pd(OAc)_2$ (500 mg, 2.23 mmol). The mixture was vacuumed and backfilled with CO twice. Then a CO balloon was installed. And the black mixture was stirred at 50 ºC for 4 h. The resulting mixture was diluted with EtOAc (300 mL), filtered, evaporated, and chromatographed with 25% EtOAc in hexanes to give ester **S4** (4.70 g, 13.8 mmol, 82%) as a white solid.

Synthesis of G-9807 intermediate **S5**:

To dry EtOAc (30 mL) was added NaH (1.40 g, 35.0 mmol). The mixture was purged with N_2 for 10 min. Then compound **S4** (2.45 g, 7.21 mmol) was added. The mixture was stirred at 90 ºC overnight. It was cooled to rt, and quenched with AcOH (15 mL). The mixture was diluted with EtOAc (60 mL), washed with H_2O (60 mL), and brine (60

mL), dried with Na_2SO_4, and evaporated. It was chromatographed with 10% EtOAc in hexanes to give crude product as a red oil which contained ethyl acetoacetate. The red oil was cooled at -20 ºC overnight. The product **S5** crystallized out, which was collected by filtration, and washed with 2:1 Et_2O:hexanes (20 mL) to give a white solid (2.40 g, 6.06 mmol, 84%).

Synthesis of G-9807 intermediate **S7**:

A mixture of compound **S5** (816 mg, 2.02 mmol) and compound **S6** (555 mg, 3.03 mmol) in dry MeOH (60 mL) was purged with N_2 for 10 min. Then a solution of NaOMe in MeOH (1.2 mL, 25wt%, 5.25 mmol) was added. The mixture was stirred at 85ºC overnight. The mixture was evaporated, and chromatographed with 5% MeOH in DCM plus 1% $NH_3 \cdot H_2O$ to give compound **S7** (176 mg, 0.371 mmol, 18%) as an off-white solid.

Synthesis of G-9807 intermediate **S9**:

To a mixture of compound **S7** (73 mg, 0.15 mmol) in MeCN (10 mL) was added aq. KOAc (1 M, 2 mL) and aq. Na_2CO_3 (1 M, 2 mL). The mixture was purged with N_2 for 10 min. Then Pd(dppf)Cl_2 (40 mg, 0.055 mmol), and boronic acid **S8** (150 mg, 0.94 mmol) was added. The mixture was stirred at 110 ºC for 3 h. The resulting mixture was concentrated, and chromatographed with 5% MeOH in DCM plus 1% $NH_3 \cdot H_2O$ to give compound **S9** (61 mg, 0.12 mmol, 78%) as a tan solid.

Synthesis of G-9807:

To a mixture of compound **S9** (72 mg, 0.14 mmol) in dioxane (12 mL) was added 4 M HCl in dioxane (12 mL). The mixture was stirred at rt for 4 h. It was then evaporated, and chromatographed with 5% MeOH in DCM plus 1% $NH_3 \cdot H_2O$ to give **G-9807** (42 mg, 0.099, 70%) as a white solid.

AMG-18 was synthesized using a previously reported procedure.[1] G-1749 and derivatives were obtained from common aniline intermediate **3Boc**, *tert*-butyl (*S*)-3-((4-(2-((5-amino-2-methylnaphthalen-1-yl)oxy)pyridin-3-yl)pyrimidin-2-yl)amino)piperidine-1-carboxylate, which was in turn synthesized using the same procedure as AMG-18 (Harrington et al., 2015). Boc deprotection of **3Boc** according to procedures described below afforded compound **3**.

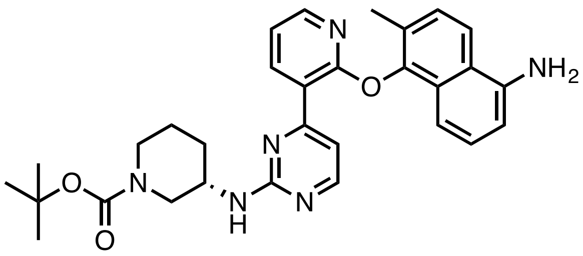

3Boc

A representative synthetic procedure for carbamate compounds (G-1749 and cpds. **2**, **7**, **8**, **9**, **11**) is reported in Supplementary Figure 13.

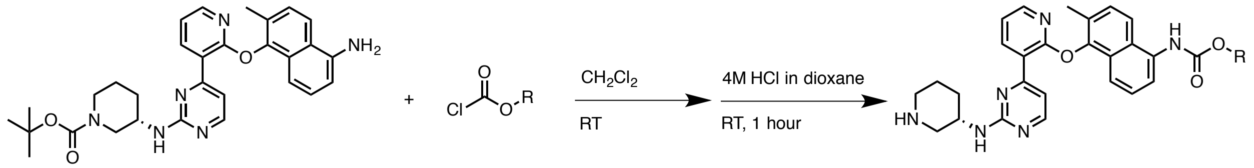

3Boc

R=
G-1749: ethyl
2: methyl
7: isopropyl
8: isobutyl
9: neopentyl
11: propyl

Supplementary Figure 13 – Synthesis of G-1749 and compounds 2, 7, 8, 9, 11.
The general method for synthesis of G-1749 and related compounds from common intermediate **3Boc** is reported.

The appropriate chloroformate (1.2 eq.) reagent was added dropwise to a solution in dry dichloromethane (2 mL) of **3Boc** (100 mg, 1 eq.). The resulting solution was left to react at room temperature and the reaction progression was followed by LC-MS. Solvent and chloroformate were evaporated under vacuum. The residue was redissolved in dry dichloromethane (2 mL) and hydrochloric acid (4 M in dioxane) (1 mL) was added slowly to the solution. After one hour, the heterogeneous mixture was evaporated to dryness under high vacuum. The crude residue was purified by C18 reverse phase HPLC (40 – 90% MeCN/10mM pH= 3.8 aqueous ammonium formate). The appropriate fractions were collected and evaporated *in vacuo* to obtain the final products as tan/white solids with yields over two steps ranging from 70% to 90%.

A representative synthetic procedure for amide compounds (G-7658 and cpds. **1**, **4**, **5**, **6**, **10**, **12**) is reported in Supplementary Figure 14.

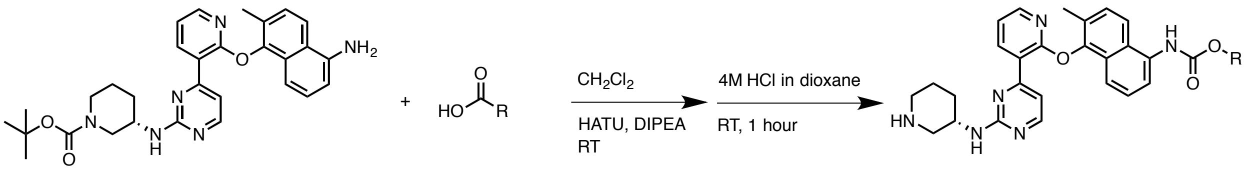

3Boc

R=
G-7658: propynyl
1: propenyl
4: methyl
5: ethyl
6: propyl
10: dimethylbutynyl
12: dimethylpentynyl

Supplementary Figure 14 – Synthesis of G-7658 and compounds 1, 4, 5, 6, 10, 12.
The general method for synthesis of G-7658 and related compounds from common intermediate **3Boc** is reported.

To a solution in dry dichloromethane (3 mL) of **3Boc** (100 mg, 1 eq.), HATU (2 eq), and the appropriate carboxylic acid (1.1 eq.), N,N-diisoethylpropylamine (2 eq.) was added dropwise. The resulting dark orange solution was left to react at room temperature and the reaction progression was followed by LC-MS. Water (5 mL) was added to the mixture, phases were separated and the aqueous phase was extracted with dichloromethane (2x5mL). Organic phases were combined, washed with brine (5 mL), dried over sodium sulfate, and evaporated to dryness. The residue was redissolved in dry dichloromethane (2 mL) and hydrochloric acid (4 M in dioxane) (1 mL) was added slowly to the solution. After two hours, the heterogeneous mixture was evaporated to dryness under high vacuum. The crude residue was purified by C18 reverse phase HPLC (40 – 90% MeCN/10mM pH= 3.8 aqueous ammonium formate). The appropriate fractions were collected and evaporated *in vacuo* to obtain the final products as tan/white solids with yields over two steps ranging from 60% to 80%.

Compounds were characterized by NMR spectroscopy and HRMS as reported below.

Compound	NMR	HRMS
**G-1749**	^{1}H NMR (500 MHz, DMSO-d_6) δ 9.57 (s, 1H), 8.48 (d, *J* = 5.2 Hz, 1H), 8.05 (dd, *J* = 4.7, 2.0 Hz, 1H), 7.93 (d, *J* = 8.7 Hz, 1H), 7.65 – 7.53 (m, 2H), 7.48 (dt, *J* = 14.1, 7.6 Hz, 2H), 7.39 (t, *J* = 7.9 Hz, 1H), 7.27 (dd, *J* = 7.6, 4.7 Hz, 1H), 4.26 (s, 1H), 4.17 (q, *J* = 7.1 Hz, 2H), 3.21 (d, *J* = 12.5 Hz, 1H), 2.86 (dd, *J* = 19.5, 10.7 Hz, 2H), 2.21 (s, 3H), 2.02 (d, *J* = 10.8 Hz, 1H), 1.91 (d, *J* = 4.3 Hz, 1H), 1.81 – 1.69 (m, 1H), 1.64 (d, *J* = 11.4 Hz, 1H), 1.28 (t, *J* = 7.1 Hz, 2H).<br><br>^{13}C NMR (126 MHz, DMSO-d_6) δ 162.11, 160.89, 159.48, 155.32, 149.13, 146.95, 140.60, 134.76, 129.11, 128.84, 128.48, 127.22, 126.64, 120.93, 120.75, 120.57, 119.40, 118.46, 111.13, 60.84, 47.16, 45.20, 43.69, 28.48, 21.05, 16.56, 15.03.	Calc. Exact Mass = 498.2379<br><br>Found = 499.2452 (MW + H^+)
**G-9807**	^{1}H NMR (400 MHz, DMSO-d_6) δ 14.24 (s, 1H), 8.89 (q, *J* = 1.7 Hz, 1H), 8.69 (q, *J* = 1.9 Hz, 1H), 7.55 (tt, *J* = 8.4, 6.5 Hz, 1H), 7.32 (t, *J* = 8.2 Hz, 2H), 6.39 (s, 1H), 4.12 (d, *J* = 13.4 Hz, 2H), 3.76 (dt, *J* = 8.3, 4.3 Hz, 2H), 1.78 (ddt, *J* = 13.1, 7.0, 3.7 Hz, 2H), 1.40 (ddt, *J* = 13.5, 8.9, 4.4 Hz, 2H).<br><br>^{13}C NMR (101 MHz, DMSO-d_6) δ 161.38, 161.31, 158.93, 158.85, 152.72, 150.40, 142.39, 133.95, 131.00, 130.90, 130.79, 119.09, 115.55, 113.46, 112.78, 112.71, 112.59, 112.52, 65.94, 42.55, 34.11.	Calc. Exact Mass = 424.1459<br><br>Found = 425.1532 (MW + H^+)
**G-7658**	^{1}H NMR (400 MHz, DMSO-d_6) δ 10.64 (s, 1H), 8.49 (d, *J* = 7.6 Hz, 1H), 8.41 (d, *J* = 5.1 Hz, 1H), 8.03 (dd, *J* = 4.8, 2.0 Hz, 1H), 7.84 (d, *J* = 8.7 Hz, 1H), 7.57 (d, *J* = 8.3 Hz, 1H), 7.52 (dd, *J* = 8.2, 3.8 Hz, 2H), 7.42 (dd, *J* = 10.3, 6.4 Hz, 2H), 7.25 (dd, *J* = 7.5, 4.8 Hz, 1H), 7.11 (d, *J* = 8.0 Hz, 1H), 3.88 (s, 1H), 3.14 – 3.04 (m, 1H), 2.78 (dt, *J* = 12.6, 4.0 Hz, 1H), 2.44 (ddd, *J* = 14.4, 11.9, 9.2 Hz, 2H), 2.22 (s, 3H), 2.10 (s, 3H), 1.97 – 1.82 (m, 1H), 1.64 (dt, *J* = 12.5, 3.9 Hz, 1H), 1.57 – 1.34 (m, 2H).<br><br>^{13}C NMR (101 MHz, DMSO-d_6) δ 162.40, 160.78, 152.23, 148.88, 146.95, 140.57, 133.29, 129.50, 128.76, 128.48, 127.47, 126.60, 122.70, 121.05, 120.60, 119.88,	Calc. Exact Mass = 492.2274<br>Found = 493.2342 (MW + H^+)

	119.48, 110.11, 85.06, 76.27, 51.89, 48.25, 46.34, 31.11, 25.67, 16.61, 3.76.	
**1**	^{1}H NMR (400 MHz, DMSO-d_6) δ 9.96 (s, 1H), 8.53 (s, 1H), 8.47 – 8.37 (m, 1H), 8.04 (dd, *J* = 4.8, 2.0 Hz, 1H), 7.92 (t, *J* = 6.4 Hz, 1H), 7.72 (d, *J* = 7.4 Hz, 1H), 7.57 – 7.45 (m, 4H), 7.41 (dd, *J* = 8.5, 7.4 Hz, 1H), 7.26 (dd, *J* = 7.6, 4.9 Hz, 2H), 6.86 (dq, *J* = 15.3, 6.8 Hz, 1H), 6.41 (d, *J* = 15.2 Hz, 1H), 4.02 (s, 1H), 3.22 (d, *J* = 11.3 Hz, 3H), 2.98 – 2.86 (m, 1H), 2.65 – 2.52 (m, 2H), 2.22 (s, 3H), 1.96 (s, 1H), 1.91 (dd, *J* = 6.9, 1.7 Hz, 3H), 1.74 (s, 1H), 1.54 (t, *J* = 9.4 Hz, 2H), 1.08 (d, *J* = 6.2 Hz, 1H).<br><br>^{13}C NMR (101 MHz, DMSO-d_6) δ 164.70, 162.27, 160.81, 148.99, 146.97, 140.61, 140.46, 134.39, 129.18, 128.73, 128.11, 127.29, 126.68, 126.30, 121.40, 120.88, 120.47, 119.47, 118.78, 110.46, 50.12, 47.12, 45.31, 30.17, 24.04, 18.06, 16.63.	Calc. Exact Mass = 494.2430<br><br>Found = 495.2503 (MW + H^+)
**2**	^{1}H NMR (400 MHz, DMSO-d_6) δ 9.59 (s, 1H), 8.55 – 8.45 (m, 1H), 8.40 (t, *J* = 5.5 Hz, 1H), 8.02 (dd, *J* = 4.8, 2.0 Hz, 1H), 7.92 (dd, *J* = 8.8, 2.7 Hz, 1H), 7.58 – 7.53 (m, 1H), 7.49 (dd, *J* = 8.8, 5.0 Hz, 2H), 7.46 – 7.41 (m, 1H), 7.41 – 7.34 (m, 1H), 7.32 – 7.20 (m, 1H), 7.14 – 7.05 (m, 1H), 3.89 (s, 1H), 3.71 (s, 3H), 3.09 (d, *J* = 11.8 Hz, 1H), 2.79 (dd, *J* = 12.2, 4.5 Hz, 1H), 2.44 (ddd, *J* = 14.8, 10.7, 8.1 Hz, 2H), 2.21 (s, 3H), 2.19 – 2.13 (m, 1H), 1.92 (d, *J* = 11.8 Hz, 1H), 1.70 – 1.58 (m, 1H), 1.56 – 1.35 (m, 2H).<br><br>^{13}C NMR (101 MHz, DMSO-d_6) δ 162.56, 161.22, 160.91, 159.25, 155.78, 148.77, 147.14, 140.40, 134.70, 129.11, 128.98, 128.61, 127.24, 126.51, 121.26, 120.94, 120.41, 119.28, 118.76, 110.20, 52.18, 51.95, 48.29, 46.44, 31.04, 25.43, 16.52.	Calc. Exact Mass = 484.2223<br><br>Found = 485.2296 (MW + H^+)
**3**	^{1}H NMR (400 MHz, DMSO-d_6) δ 8.49 (d, *J* = 7.3 Hz, 1H), 8.40 (d, *J* = 5.1 Hz, 1H), 8.01 (dd, *J* = 4.8, 2.0 Hz, 1H), 7.92 (d, *J* = 8.7 Hz, 1H), 7.44 (d, *J* = 5.1 Hz, 1H), 7.30 (d, *J* = 8.7 Hz, 1H), 7.22 (dd, *J* = 7.6, 4.8 Hz, 1H), 7.17 – 7.10 (m, 1H), 7.07 (d, *J* = 7.9 Hz, 1H), 6.81 (d, *J* = 8.3 Hz, 1H), 6.60 (dd, *J* = 7.5, 1.0 Hz, 1H), 5.77 (s, 2H), 3.91 (s, 1H), 3.16 – 3.06 (m, 1H), 2.82 (dd, *J* = 12.4, 4.3 Hz, 1H), 2.48 – 2.40 (m, 2H), 2.18 (s, 3H),	Calc. Exact Mass = 426.2168<br><br>Found = 427.2241 (MW + H^+)

	1.99 – 1.88 (m, 1H), 1.72 – 1.61 (m, 1H), 1.47 (qd, *J* = 11.2, 9.7, 3.8 Hz, 2H).<br><br>^{13}C NMR (101 MHz, DMSO-d_6) δ 162.38, 160.97, 148.92, 146.86, 145.54, 140.39, 129.08, 127.81, 126.73, 126.57, 123.19, 120.84, 120.13, 119.18, 110.19, 108.90, 107.46, 51.52, 48.00, 46.12, 30.91, 25.33, 16.65.	
**4**	^{1}H NMR (400 MHz, DMSO-d_6) δ 9.96 (d, *J* = 8.4 Hz, 1H), 8.46 (d, *J* = 5.1 Hz, 1H), 8.05 (dd, *J* = 4.6, 2.0 Hz, 1H), 7.95 (d, *J* = 8.6 Hz, 1H), 7.63 (d, *J* = 7.6 Hz, 1H), 7.57 – 7.44 (m, 3H), 7.44 – 7.32 (m, 2H), 7.26 (dd, *J* = 7.6, 4.7 Hz, 1H), 4.17 (s, 1H), 3.50 (dt, *J* = 18.1, 5.5 Hz, 1H), 3.15 – 3.02 (m, 1H), 2.75 (td, *J* = 12.7, 12.2, 9.1 Hz, 2H), 2.22 (s, 3H), 2.20 (s, 3H), 2.17 (s, 1H), 2.10 – 1.95 (m, 2H), 1.85 (dt, *J* = 13.1, 4.2 Hz, 1H), 1.73 – 1.56 (m, 2H).<br><br>^{13}C NMR (101 MHz, DMSO) δ 169.43, 162.34, 161.23, 160.93, 159.36, 148.95, 148.81, 147.14, 140.49, 134.79, 129.04, 128.50, 127.18, 126.48, 121.53, 120.52, 120.46, 119.32, 118.83, 110.79, 109.64, 49.06, 46.42, 44.79, 29.47, 23.79, 22.67, 19.85, 16.54.	Calc. Exact Mass = 468.2274<br><br>Found = 469.2347 (MW + H^+)
**5**	^{1}H NMR (400 MHz, DMSO-d_6) δ 9.89 (s, 1H), 8.56 – 8.44 (m, 1H), 8.41 (d, *J* = 5.0 Hz, 1H), 8.03 (dd, *J* = 4.8, 1.9 Hz, 1H), 7.92 (d, *J* = 8.7 Hz, 1H), 7.62 (d, *J* = 7.3 Hz, 1H), 7.50 (d, *J* = 8.8 Hz, 2H), 7.46 – 7.35 (m, 2H), 7.25 (dd, *J* = 7.5, 4.8 Hz, 1H), 7.10 (d, *J* = 8.0 Hz, 1H), 3.83 (d, *J* = 36.8 Hz, 2H), 3.08 (d, *J* = 8.4 Hz, 1H), 2.78 (d, *J* = 12.1 Hz, 1H), 2.48 – 2.35 (m, 2H), 2.22 (s, 3H), 1.90 (t, *J* = 14.6 Hz, 1H), 1.64 (d, *J* = 8.5 Hz, 1H), 1.58 – 1.36 (m, 2H), 1.17 (t, *J* = 7.5 Hz, 3H).<br><br>^{13}C NMR (126 MHz, DMSO) δ 173.19, 162.40, 160.81, 148.90, 146.97, 140.55, 134.56, 129.11, 128.76, 128.35, 127.24, 126.67, 121.60, 121.04, 120.56, 119.44, 118.75, 110.11, 51.94, 48.28, 46.37, 31.14, 29.54, 25.72, 16.63, 10.37.	Calc. Exact Mass = 482.2430<br><br>Found = 483.2500 (MW + H^+)
**6**	^{1}H NMR (400 MHz, DMSO-d_6) δ 9.89 (s, 1H), 8.50 (d, *J* = 7.7 Hz, 1H), 8.41 (d, *J* = 5.2 Hz, 1H), 8.03 (dd, *J* = 4.6, 2.0 Hz, 1H), 7.91 (d, *J* = 8.6 Hz, 1H), 7.61 (d, *J* = 7.5 Hz, 1H), 7.51 (d, *J* = 8.6 Hz, 2H), 7.45 (d, *J* = 5.3 Hz, 1H), 7.40 (t, *J* = 8.0 Hz, 1H), 7.25 (dd, *J* = 7.5, 4.7 Hz, 1H), 7.14 (d, *J* = 8.0 Hz, 1H), 3.92 (s, 1H), 3.18 – 3.05 (m, 1H), 2.82 (dt, *J* = 12.3, 4.0 Hz, 1H), 2.22 (s,	Calc. Exact Mass = 496.2587<br><br>Found = 497.2660 (MW + H^+)

	3H), 1.98 – 1.87 (m, 1H), 1.68 (dq, *J* = 13.6, 6.5, 5.6 Hz, 3H), 1.48 (tdd, *J* = 13.6, 9.9, 3.5 Hz, 2H), 0.99 (t, *J* = 7.4 Hz, 3H).<br><br>^{13}C NMR (101 MHz, DMSO-d_6) δ 172.31, 162.36, 160.80, 148.91, 146.96, 140.55, 134.51, 129.13, 128.75, 128.38, 127.23, 126.65, 121.75, 120.99, 120.52, 119.44, 118.80, 110.19, 51.44, 47.97, 46.07, 38.33, 30.88, 25.27, 19.28, 16.63, 14.18.	
**7**	^{1}H NMR (400 MHz, DMSO-d_6) δ 9.49 (s, 1H), 8.49 (d, *J* = 7.1 Hz, 1H), 8.41 (d, *J* = 5.1 Hz, 1H), 8.02 (dd, *J* = 4.8, 2.0 Hz, 1H), 7.95 – 7.88 (m, 1H), 7.55 (dd, *J* = 7.4, 1.2 Hz, 1H), 7.48 (dd, *J* = 8.8, 3.8 Hz, 2H), 7.44 (d, *J* = 5.1 Hz, 1H), 7.39 (dd, *J* = 8.5, 7.4 Hz, 1H), 7.25 (dd, *J* = 7.6, 4.8 Hz, 1H), 7.11 (d, *J* = 8.0 Hz, 1H), 4.93 (p, *J* = 6.3 Hz, 1H), 3.88 (s, 1H), 3.09 (d, *J* = 11.7 Hz, 1H), 2.78 (d, *J* = 12.0 Hz, 1H), 2.47 – 2.37 (m, 2H), 2.21 (s, 3H), 1.92 (s, 1H), 1.64 (d, *J* = 11.1 Hz, 1H), 1.46 (dt, *J* = 21.6, 12.3 Hz, 2H), 1.29 (d, *J* = 6.3 Hz, 6H).<br><br>^{13}C NMR (101 MHz, DMSO) δ 162.39, 160.81, 154.93, 148.89, 146.89, 140.54, 134.70, 129.09, 128.75, 128.35, 127.23, 126.70, 120.92, 120.58, 119.43, 118.43, 110.11, 68.15, 51.87, 48.23, 46.33, 31.09, 25.65, 22.50, 16.59.	Calc. Exact Mass = 512.2536<br><br>Found = 513.2605 (MW + H^+)
**8**	^{1}H NMR (400 MHz, DMSO-d_6) δ 9.56 (s, 1H), 8.50 (d, *J* = 7.5 Hz, 1H), 8.41 (d, *J* = 5.1 Hz, 1H), 8.03 (dd, *J* = 4.8, 2.0 Hz, 1H), 7.93 (d, *J* = 8.7 Hz, 1H), 7.54 (dd, *J* = 7.3, 1.2 Hz, 1H), 7.50 (dd, *J* = 8.7, 3.8 Hz, 2H), 7.47 – 7.41 (m, 1H), 7.41 – 7.36 (m, 1H), 7.29 – 7.20 (m, 1H), 7.10 (d, *J* = 8.0 Hz, 1H), 3.92 (d, *J* = 6.7 Hz, 3H), 3.09 (d, *J* = 11.7 Hz, 1H), 2.78 (d, *J* = 12.3 Hz, 1H), 2.43 (ddd, *J* = 14.5, 12.2, 9.5 Hz, 2H), 1.95 (tt, *J* = 9.8, 5.4 Hz, 2H), 1.64 (d, *J* = 12.9 Hz, 1H), 1.56 – 1.34 (m, 2H), 0.96 (d, *J* = 6.8 Hz, 7H).<br><br>^{13}C NMR (101 MHz, DMSO) δ 162.40, 160.80, 155.49, 148.88, 146.92, 140.55, 134.62, 129.15, 128.76, 128.44, 127.28, 126.71, 121.09, 120.52, 119.44, 118.58, 110.10, 70.80, 51.91, 48.27, 46.34, 31.12, 28.12, 25.70, 19.41, 16.60.	Calc. Exact Mass = 526.2692<br><br>Found = 527.2765 (MW + H^+)
**9**	^{1}H NMR (400 MHz, DMSO-d_6) δ 9.53 (s, 1H), 8.50 (d, *J* = 7.1 Hz, 1H), 8.41 (d, *J* = 5.1 Hz, 1H), 8.03 (dd, *J* = 4.8, 2.0 Hz, 1H), 7.98 – 7.89 (m, 1H), 7.58 – 7.47 (m, 3H), 7.47 – 7.35 (m, 2H), 7.26 (dd, *J* = 7.5, 4.8 Hz, 1H),	Calc. Exact Mass = 540.2849<br><br>Found = 541.2922 (MW + H^+)

	7.11 (d, J = 8.0 Hz, 1H), 3.89 (s, 1H), 3.84 (s, 2H), 3.09 (d, J = 11.4 Hz, 1H), 2.83 – 2.74 (m, 1H), 2.44 (ddd, J = 14.2, 12.1, 9.3 Hz, 2H), 2.22 (s, 3H), 1.99 – 1.87 (m, 1H), 1.64 (dt, J = 12.4, 3.8 Hz, 1H), 1.57 – 1.37 (m, 2H), 0.97 (s, 10H).<br><br>^{13}C NMR (101 MHz, DMSO) δ 162.40, 160.79, 155.62, 148.88, 146.93, 140.55, 134.63, 129.15, 128.76, 128.52, 127.30, 126.70, 121.26, 120.53, 119.44, 118.64, 110.10, 73.86, 51.92, 48.27, 46.35, 31.95, 31.12, 26.72, 25.70, 16.61.	
**10**	^{1}H NMR (400 MHz, DMSO-d_6) δ 10.59 (s, 1H), 8.50 (d, J = 7.8 Hz, 1H), 8.41 (d, J = 5.1 Hz, 1H), 8.04 (dd, J = 4.8, 2.1 Hz, 1H), 7.81 (d, J = 8.7 Hz, 1H), 7.64 – 7.35 (m, 5H), 7.26 (dd, J = 7.6, 4.8 Hz, 1H), 7.12 (d, J = 8.0 Hz, 1H), 3.89 (s, 1H), 3.17 – 3.02 (m, 1H), 2.79 (d, J = 12.4 Hz, 1H), 2.49 – 2.37 (m, 2H), 2.22 (s, 3H), 1.92 (t, J = 7.6 Hz, 1H), 1.72 – 1.59 (m, 1H), 1.56 – 1.39 (m, 2H), 1.35 (s, 8H), 1.30 – 1.15 (m, 1H), 0.85 (s, 1H).<br><br>^{13}C NMR (126 MHz, DMSO) δ 162.39, 160.78, 152.41, 148.89, 146.95, 140.58, 133.30, 129.50, 128.76, 128.68, 127.47, 126.62, 122.99, 120.75, 120.02, 119.49, 110.11, 95.36, 75.40, 51.85, 48.22, 46.31, 31.08, 30.42, 29.66, 28.81, 27.63, 25.63, 16.62.	Calc. Exact Mass = 534.2743<br><br>Found = 535.2816 (MW + H^+)
**11**	^{1}H NMR (400 MHz, DMSO-d_6) δ 9.56 (s, 1H), 8.53 (s, 1H), 8.44 (d, J = 5.1 Hz, 1H), 8.03 (dd, J = 4.8, 2.0 Hz, 1H), 7.93 (d, J = 8.7 Hz, 1H), 7.55 (dd, J = 7.4, 1.2 Hz, 1H), 7.52 – 7.44 (m, 3H), 7.39 (dd, J = 8.5, 7.3 Hz, 1H), 7.26 (dd, J = 7.6, 4.9 Hz, 2H), 4.08 (t, J = 6.7 Hz, 3H), 2.95 (d, J = 11.9 Hz, 1H), 2.60 (ddd, J = 21.3, 10.0, 7.3 Hz, 2H), 2.21 (s, 3H), 1.96 (s, 1H), 1.80 – 1.73 (m, 1H), 1.68 (h, J = 7.2 Hz, 2H), 1.55 (t, J = 9.2 Hz, 2H), 0.96 (t, J = 7.4 Hz, 3H).<br><br>^{13}C NMR (101 MHz, DMSO) δ 162.26, 160.82, 155.43, 149.00, 146.89, 140.61, 129.15, 128.74, 128.39, 127.27, 126.73, 120.99, 120.86, 120.56, 119.46, 118.48, 110.49, 66.38, 49.93, 47.00, 45.20, 30.07, 23.85, 22.45, 16.60, 10.74.	Calc. Exact Mass = 512.2536<br><br>Found = 513.2609 (MW + H^+)
**12**	^{1}H NMR (400 MHz, DMSO-d_6) δ 10.63 (s, 1H), 8.49 (d, J = 7.7 Hz, 1H), 8.41 (d, J = 5.2 Hz, 1H), 8.03 (p, J = 2.3 Hz, 1H), 7.82 (d, J = 8.7 Hz, 1H), 7.58 (d, J = 8.5 Hz, 1H), 7.52 (dd, J = 8.1, 3.5 Hz, 2H), 7.48 – 7.35 (m, 2H), 7.26 (dd, J = 7.6, 4.8 Hz, 1H), 7.11 (d, J = 8.0 Hz,	Calc. Exact Mass = 548.2900<br><br>Found = 549.2973 (MW + H^+)

	1H), 3.88 (s, 1H), 3.09 (d, *J* = 11.9 Hz, 1H), 2.79 (dt, *J* = 12.3, 3.9 Hz, 1H), 2.47 – 2.30 (m, 4H), 2.22 (s, 3H), 2.04 – 1.85 (m, 1H), 1.64 (dt, *J* = 12.4, 4.0 Hz, 1H), 1.55 – 1.35 (m, 2H), 1.07 (s, 9H), 0.99 – 0.80 (m, 1H), 0.55 (s, 1H).<br><br>^{13}C NMR (101 MHz, DMSO) δ 162.56, 161.22, 160.88, 159.25, 148.76, 147.19, 140.41, 133.71, 129.47, 129.01, 127.44, 126.39, 121.28, 120.60, 120.25, 119.32, 110.18, 51.98, 48.32, 46.46, 31.33, 31.05, 30.26, 29.20, 25.46, 16.54.	

Supplementary References

1. Harrington, P. E. *et al.* Unfolded Protein Response in Cancer: IRE1α Inhibition by Selective Kinase Ligands Does Not Impair Tumor Cell Viability. *ACS Med. Chem. Lett.* **6,** 68–72 (2015).
