# Supplementary material for: Activation of the IRE1 RNase through remodeling of the kinase front pocket by ATP-competitive ligands
Source: Nat Commun. 2020 Dec 14;11:6387. doi: 10.1038/s41467-020-19974-5 (PMC7736581; doi:10.1038/s41467-020-19974-5)
Supplement: Supplementary file 3 — Description of Additional Supplementary Files [file 41467_2020_19974_MOESM3_ESM.docx]

File Name: Supplementary Data 1

Description: Kinase selectivity data. Results from kinome inhibition assays run at Invitrogen for G-1749, G-7658, and G-9807.

File Name: Supplementary Data 2

Description: Hydrogen-Deuterium Exchange (HX-MS) deuteration plots.

File Name: Supplementary Data 3

Description: Peptide protection factors calculated from Hydrogen-Deuterium Exchange (HX-MS) experiments.

File Name: Supplementary Data 4

Description: HX-MS Information Table.

File Name: Supplementary Data 5

Description: Phosphorylation mapping results (output of the proteomics search engine).
