# Supplementary material for: Activation of the IRE1 RNase through remodeling of the kinase front pocket by ATP-competitive ligands
Source: Nat Commun. 2020 Dec 14;11:6387. doi: 10.1038/s41467-020-19974-5 (PMC7736581; doi:10.1038/s41467-020-19974-5)
Supplement: Supplementary file 5 — Supplementary Data 2 [file 41467_2020_19974_MOESM5_ESM.pdf]

Supplementary Information
Hydrogen-Deuterium exchange (HX-MS) deuteration plots

Note: human IRE1 residue numbering is significantly offset from the sequential numbering used in the peptide plots and tables.
For reference, residue 16 in HX data is equivalent to residue 562 in the PDB files referenced in the main text. (See Figure S1A)

Legend for plot colors:

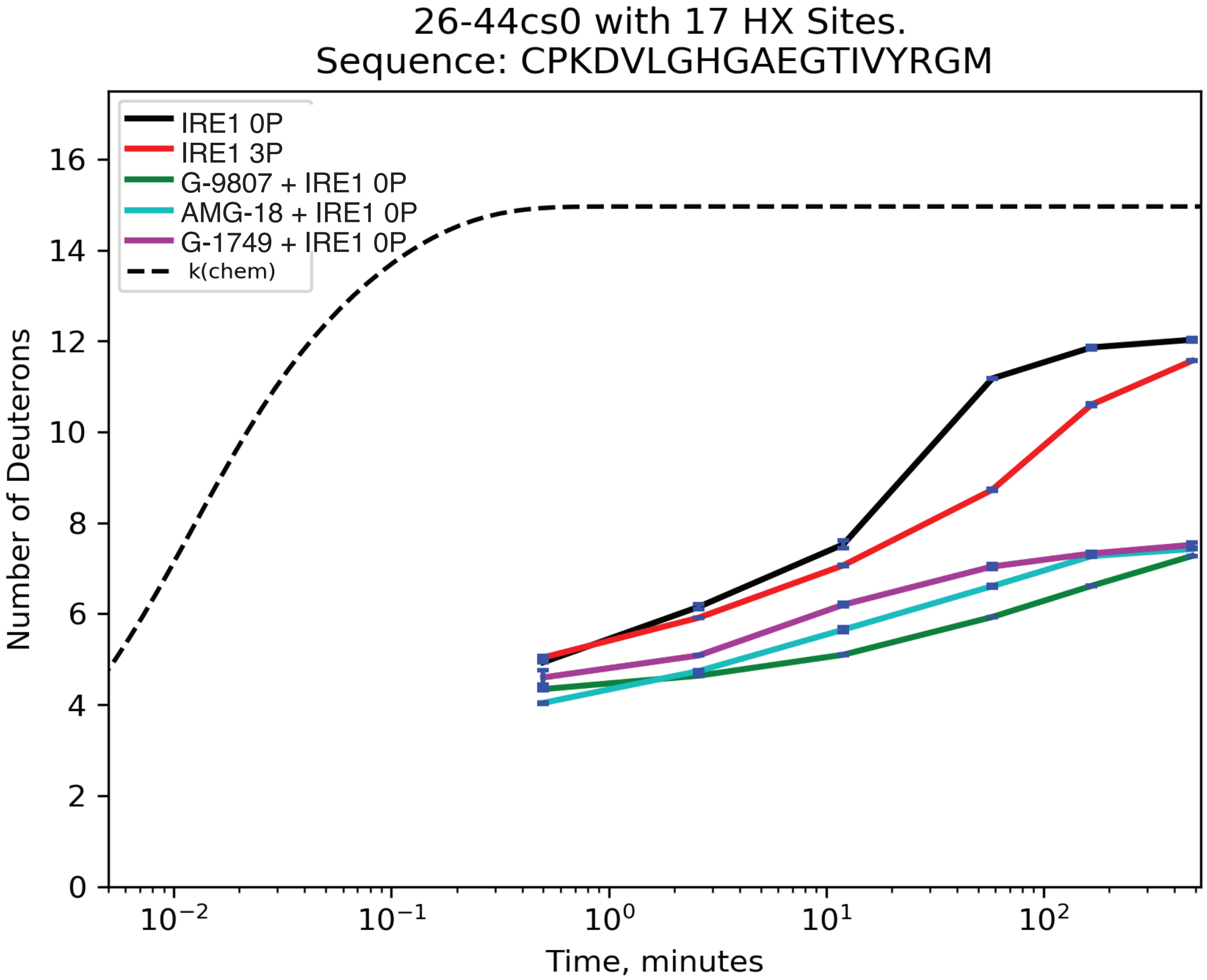

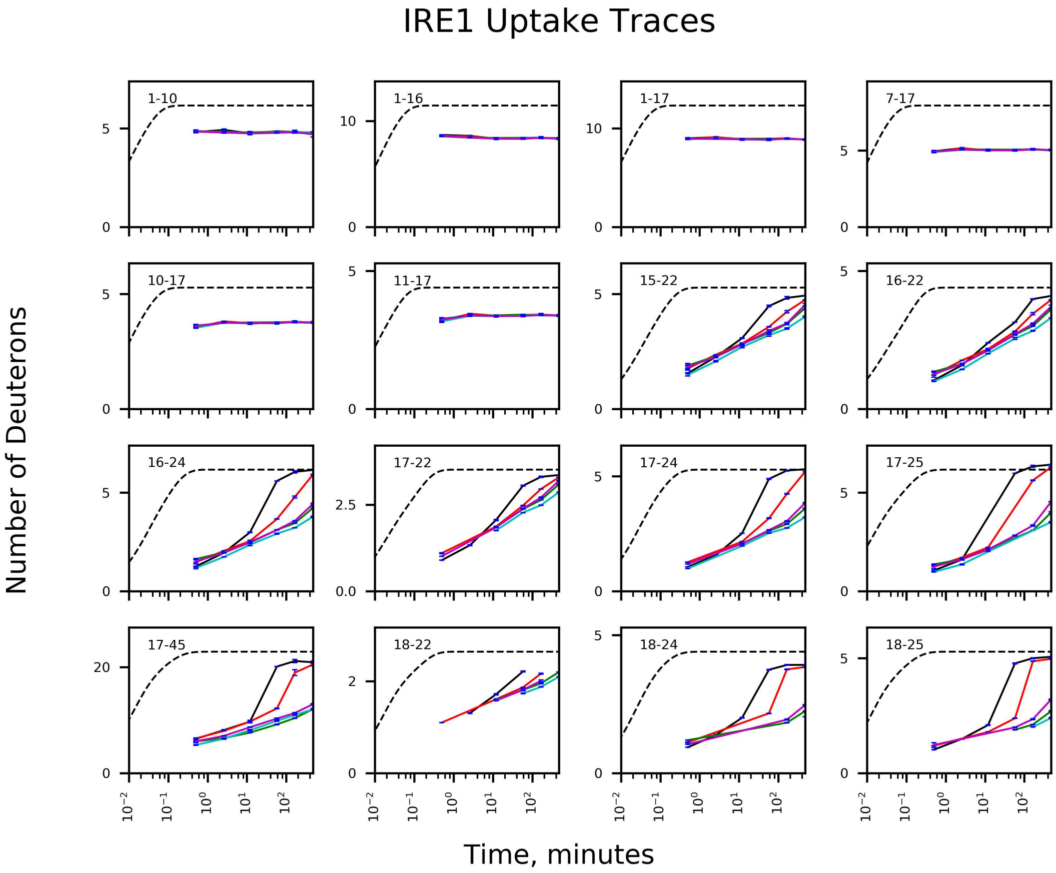

IRE1 Uptake Traces
Number of Deuterons
Time, minutes
1-10
1-16
1-17
7-17
10-17
11-17
15-22
16-22
16-24
17-22
17-24
17-25
17-45
18-22
18-24
18-25

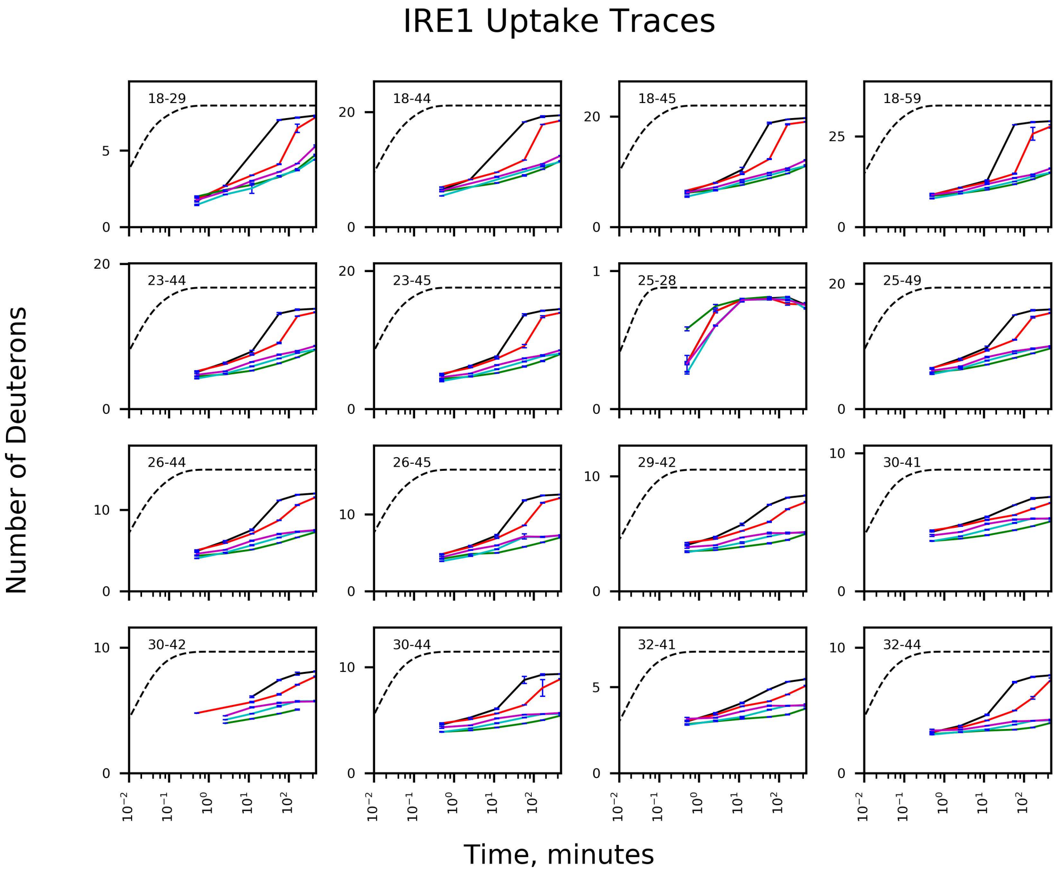
IRE1 Uptake Traces
18-29
18-44
18-45
18-59
23-44
23-45
25-28
25-49
26-44
26-45
29-42
30-41
30-42
30-44
32-41
32-44
Number of Deuterons
Time, minutes

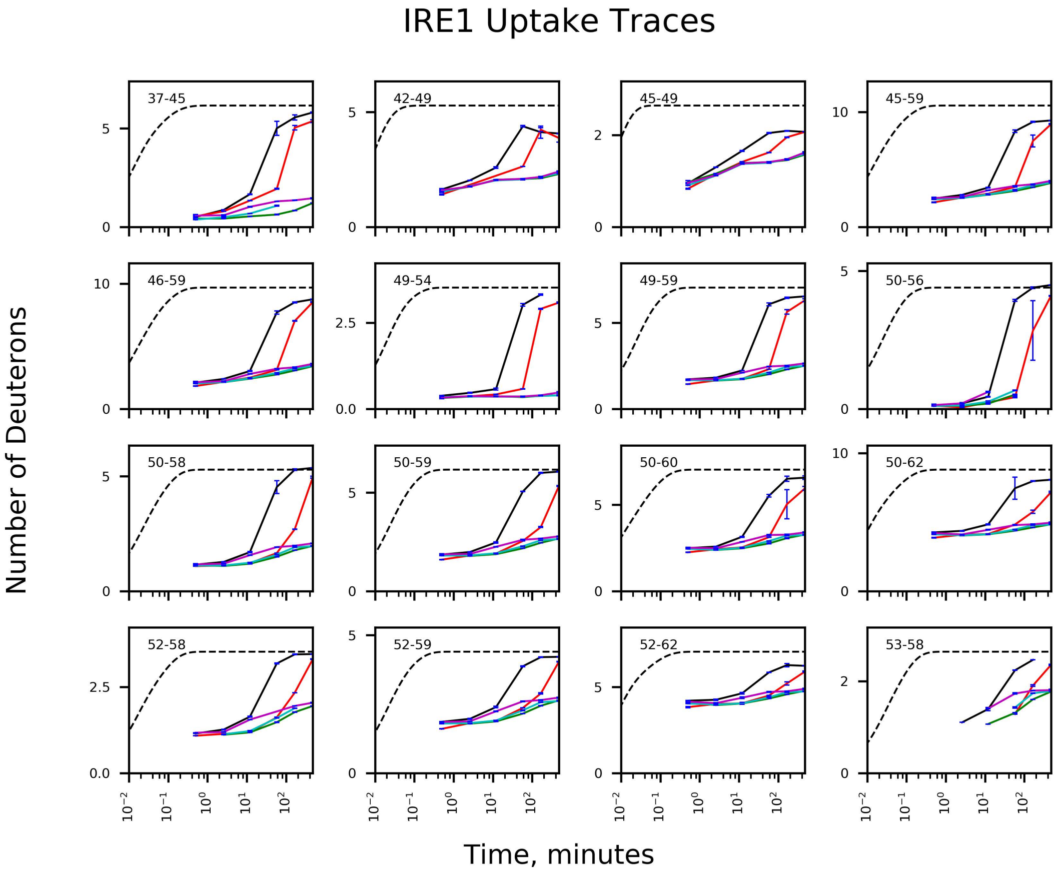

IRE1 Uptake Traces
Number of Deuterons
Time, minutes
37-45
42-49
45-49
45-59
46-59
49-54
49-59
50-56
50-58
50-59
50-60
50-62
52-58
52-59
52-62
53-58

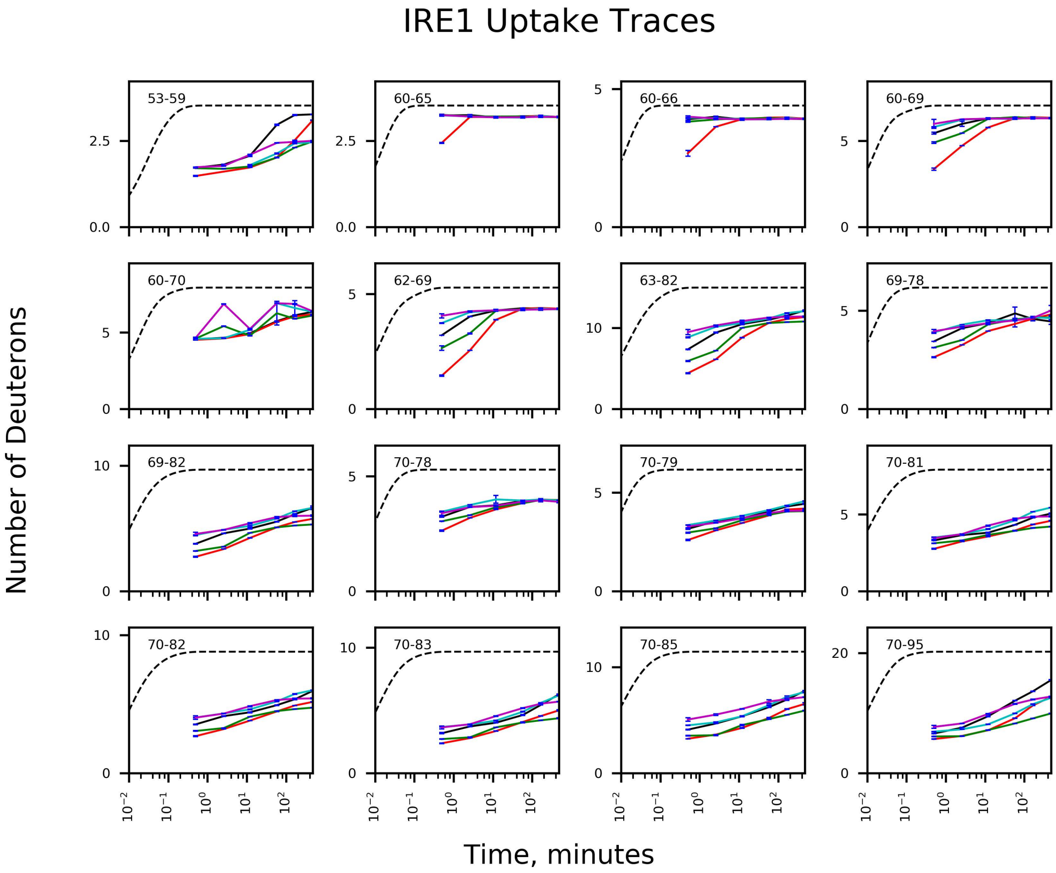

IRE1 Uptake Traces
53-59
60-65
60-66
60-69
60-70
62-69
63-82
69-78
69-82
70-78
70-79
70-81
70-82
70-83
70-85
70-95
Number of Deuterons
Time, minutes

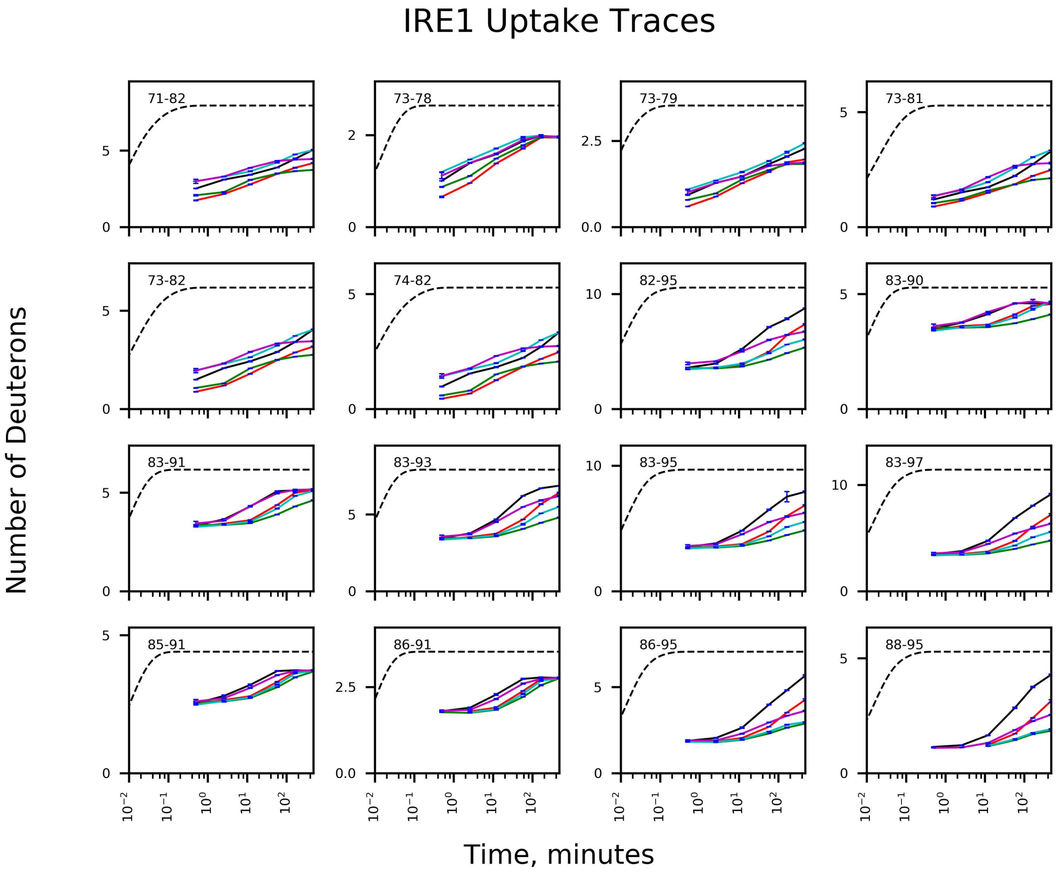
IRE1 Uptake Traces
71-82
73-78
73-79
73-81
73-82
74-82
82-95
83-90
83-91
83-93
83-95
83-97
85-91
86-91
86-95
88-95
Number of Deuterons
Time, minutes

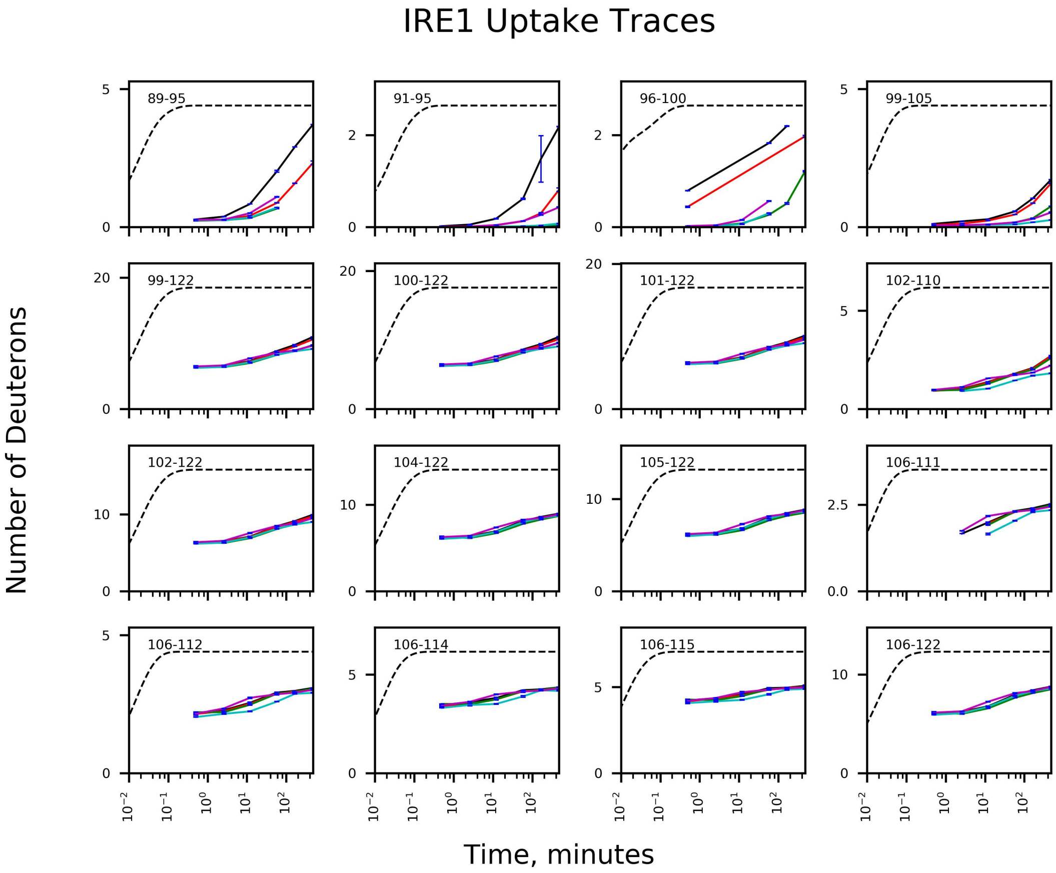
IRE1 Uptake Traces
89-95
91-95
96-100
99-105
99-122
100-122
101-122
102-110
102-122
104-122
105-122
106-111
106-112
106-114
106-115
106-122
Number of Deuterons
Time, minutes

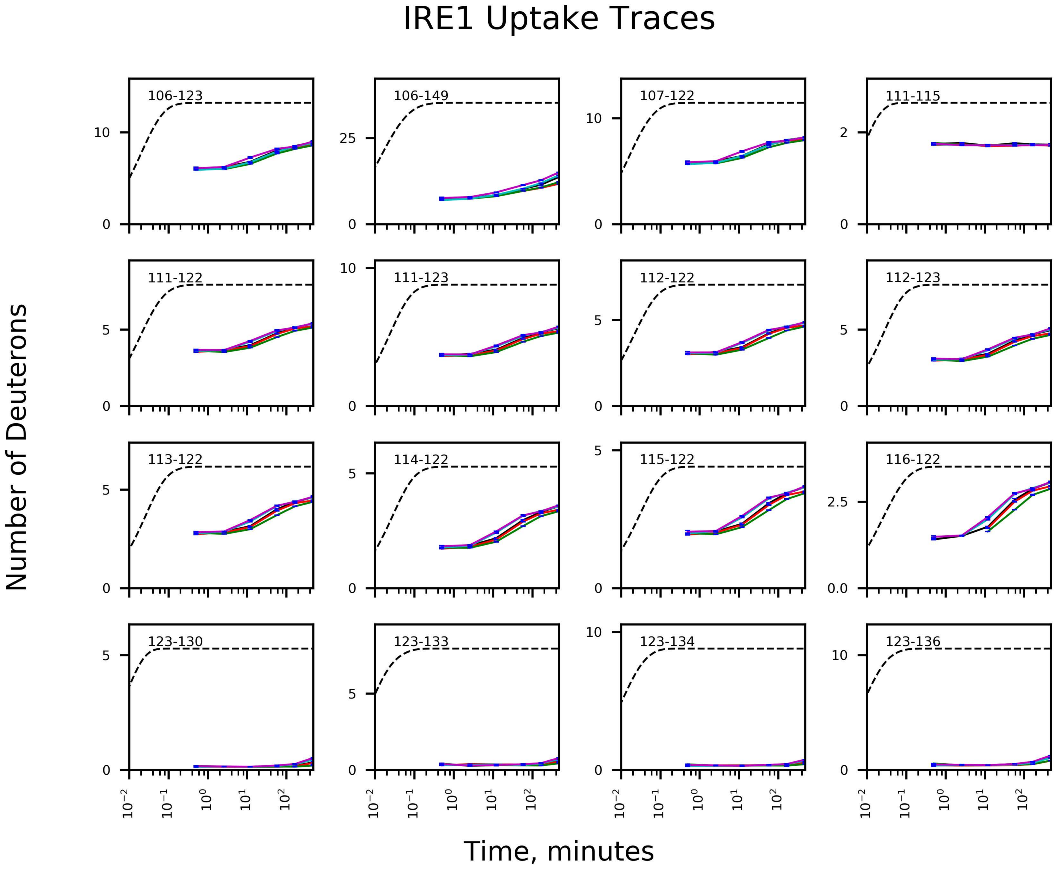

IRE1 Uptake Traces
106-123
106-149
107-122
111-115
111-122
111-123
112-122
112-123
113-122
114-122
115-122
116-122
123-130
123-133
123-134
123-136
Number of Deuterons
Time, minutes

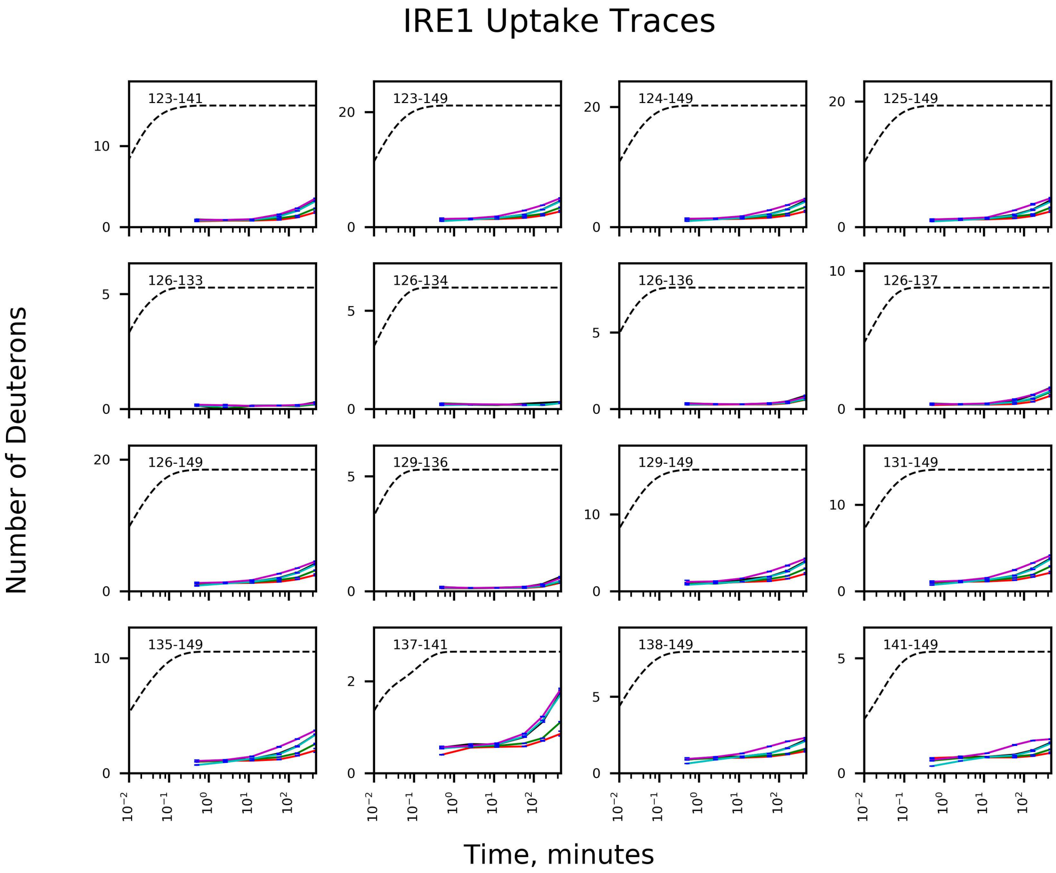

IRE1 Uptake Traces
123-141
123-149
124-149
125-149
126-133
126-134
126-136
126-137
126-149
129-136
129-149
131-149
135-149
137-141
138-149
141-149
Number of Deuterons
Time, minutes

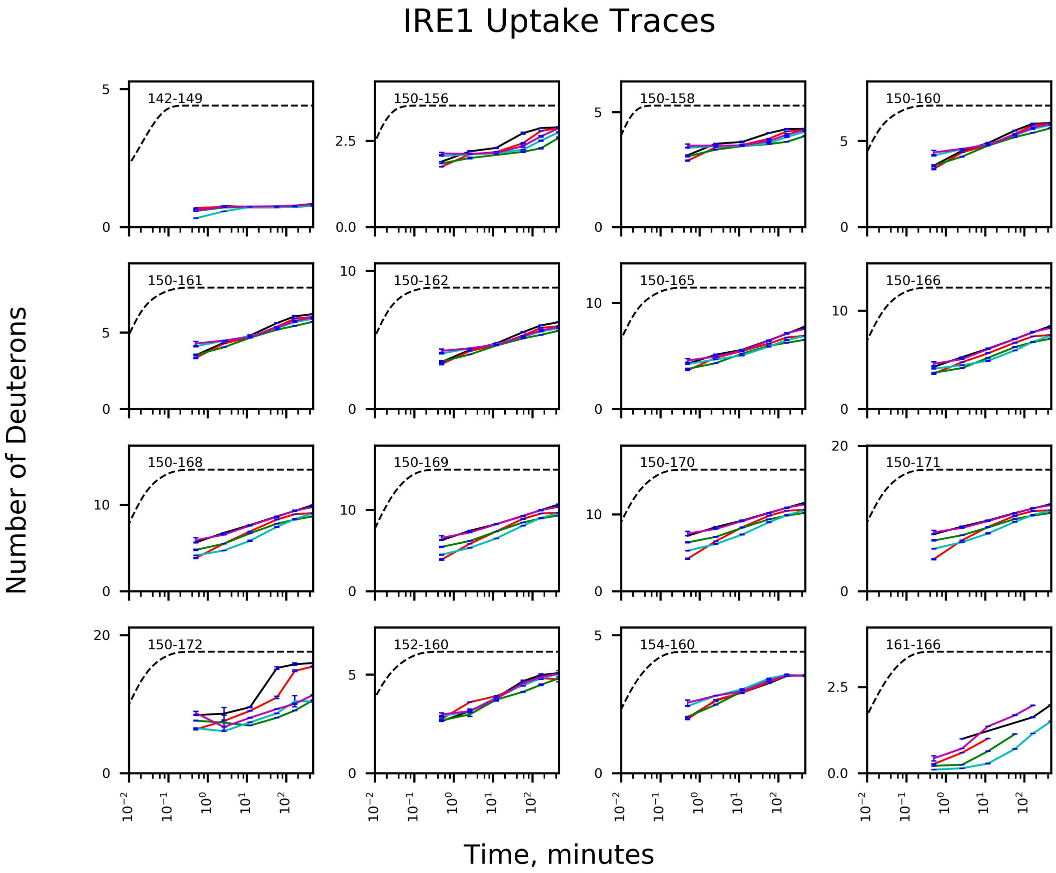

IRE1 Uptake Traces
142-149
150-156
150-158
150-160
150-161
150-162
150-165
150-166
150-168
150-169
150-170
150-171
150-172
152-160
154-160
161-166
Number of Deuterons
Time, minutes

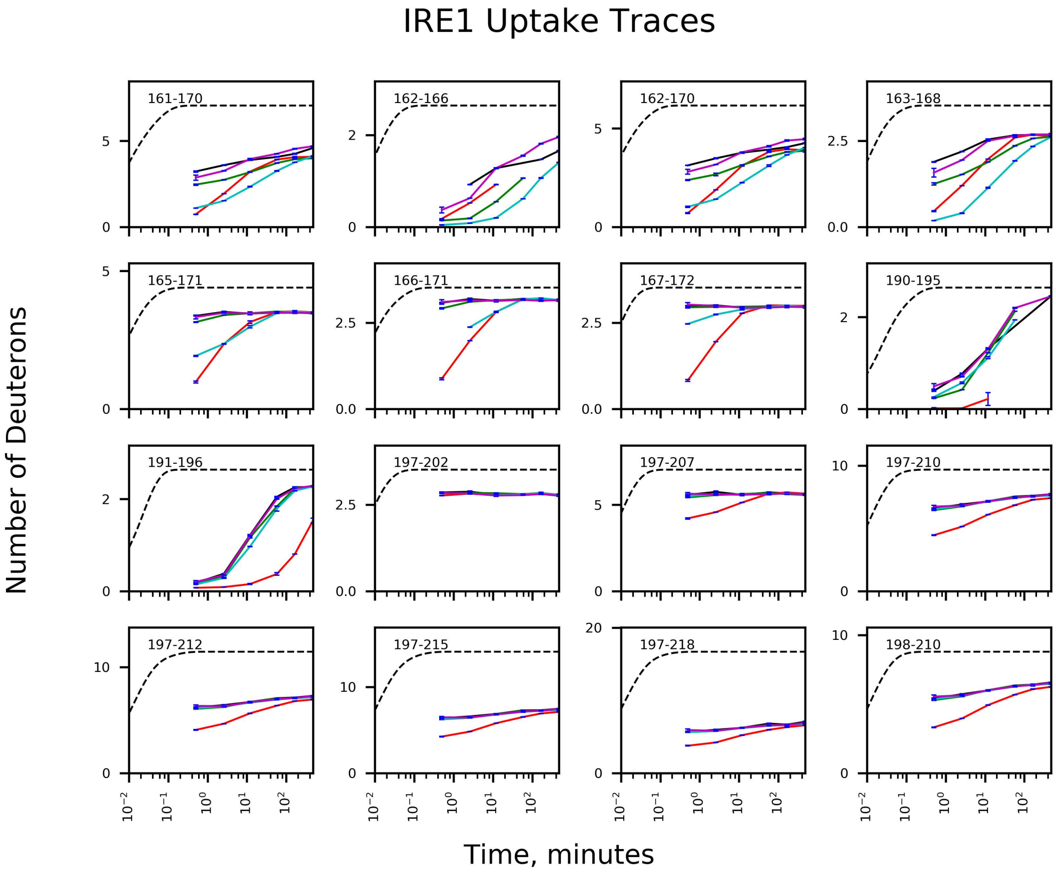
IRE1 Uptake Traces
161-170
162-166
162-170
163-168
165-171
166-171
167-172
190-195
191-196
197-202
197-207
197-210
197-212
197-215
197-218
198-210
Number of Deuterons
Time, minutes

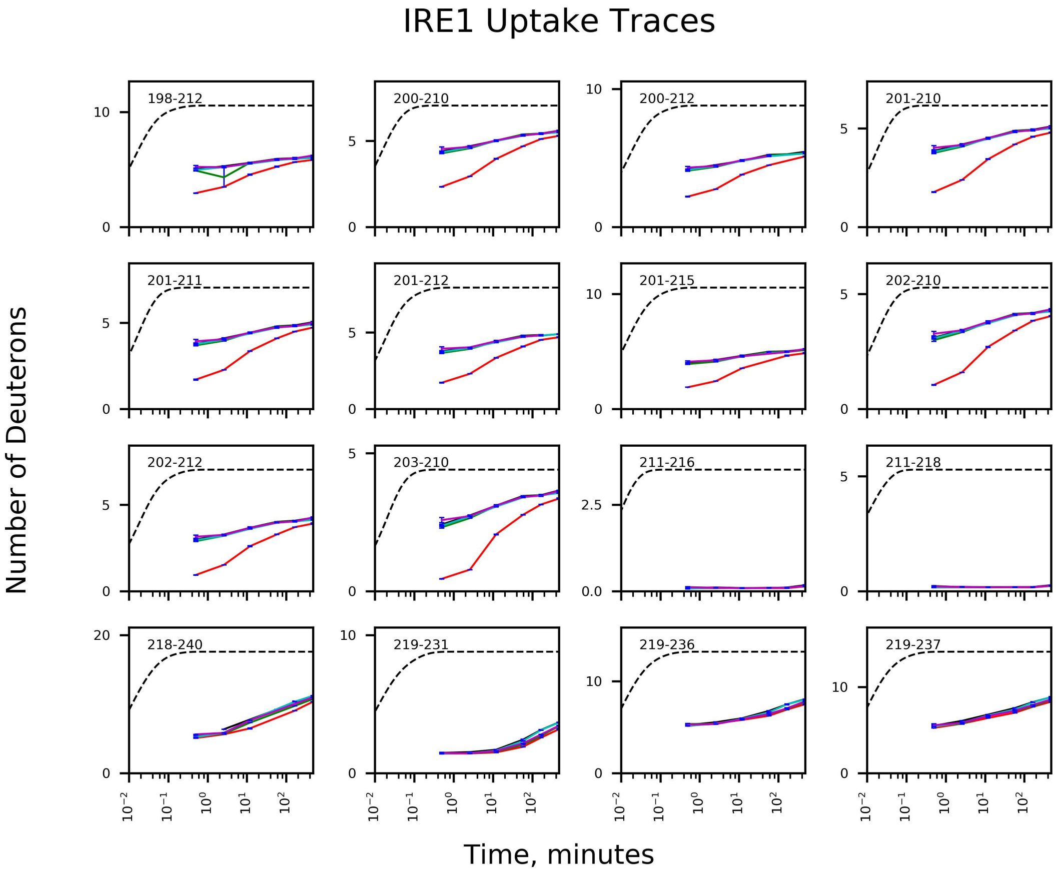
IRE1 Uptake Traces
198-212
200-210
200-212
201-210
201-211
201-212
201-215
202-210
202-212
203-210
211-216
211-218
218-240
219-231
219-236
219-237
Number of Deuterons
Time, minutes

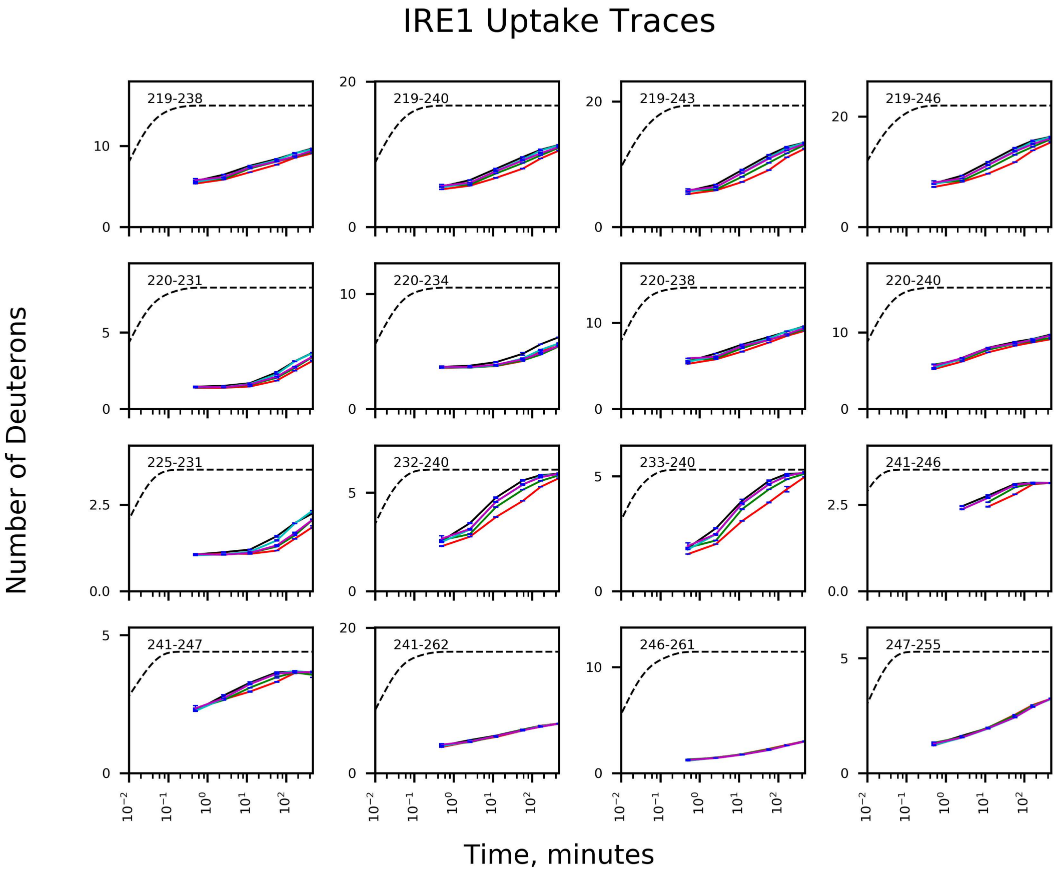

IRE1 Uptake Traces
219-238
219-240
219-243
219-246
220-231
220-234
220-238
220-240
225-231
232-240
233-240
241-246
241-247
241-262
246-261
247-255
Number of Deuterons
Time, minutes

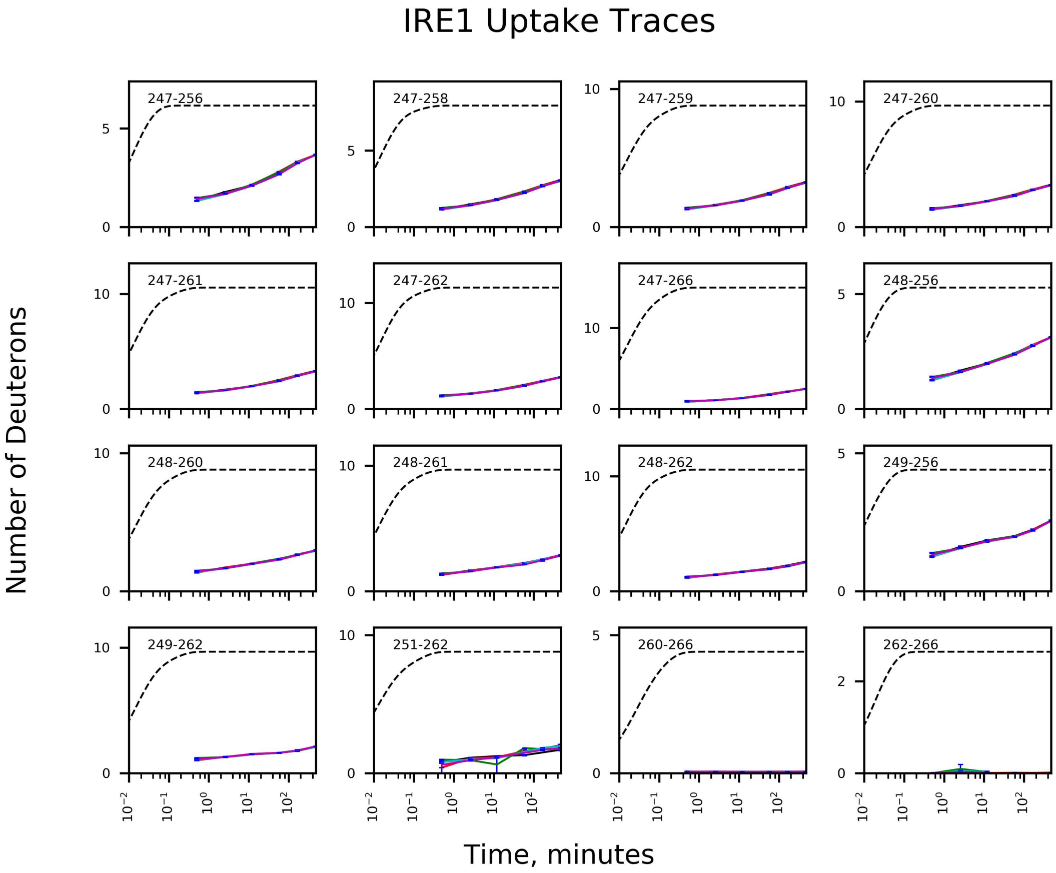
IRE1 Uptake Traces
247-256
247-258
247-259
247-260
247-261
247-262
247-266
248-256
248-260
248-261
248-262
249-256
249-262
251-262
260-266
262-266
Number of Deuterons
Time, minutes

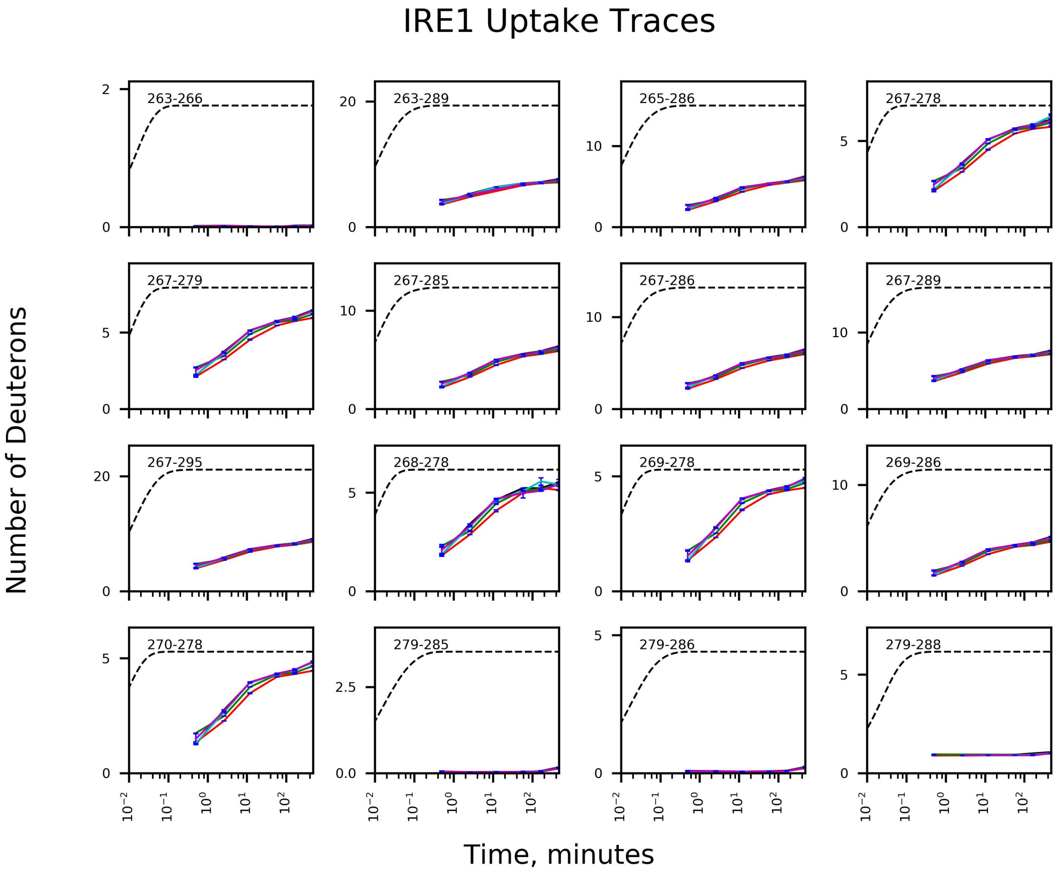
IRE1 Uptake Traces
Number of Deuterons
Time, minutes
263-266
263-289
265-286
267-278
267-279
267-285
267-286
267-289
267-295
268-278
269-278
269-286
270-278
279-285
279-286
279-288

IRE1 Uptake Traces

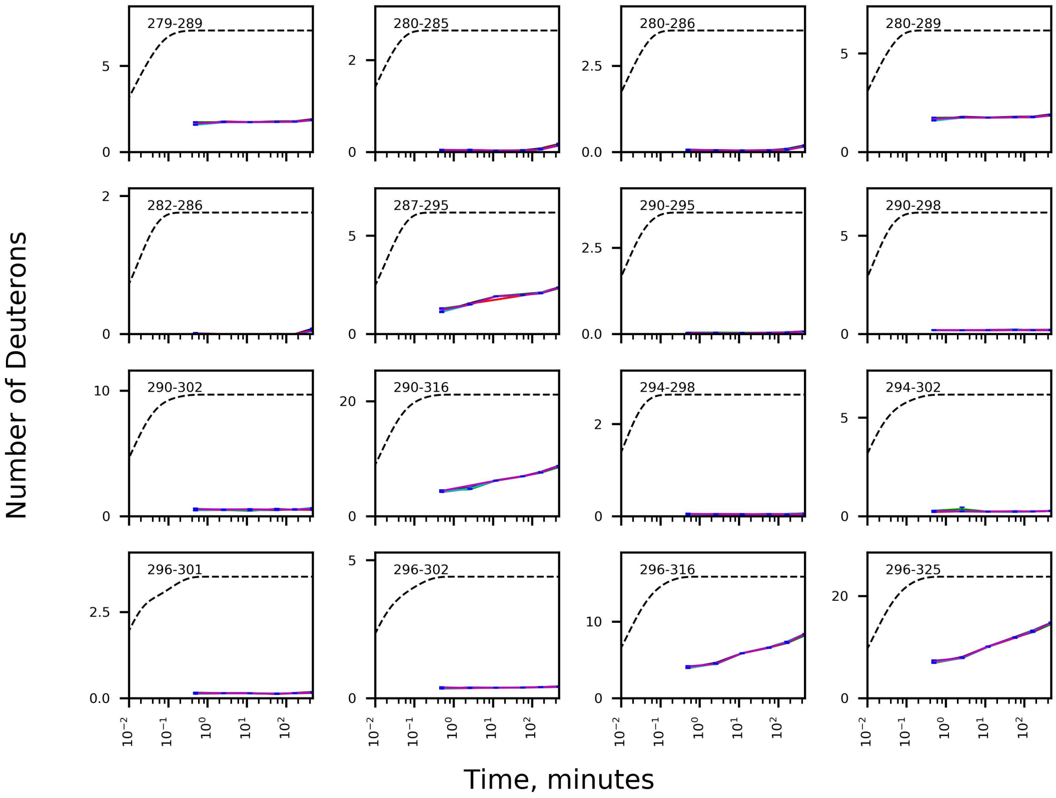

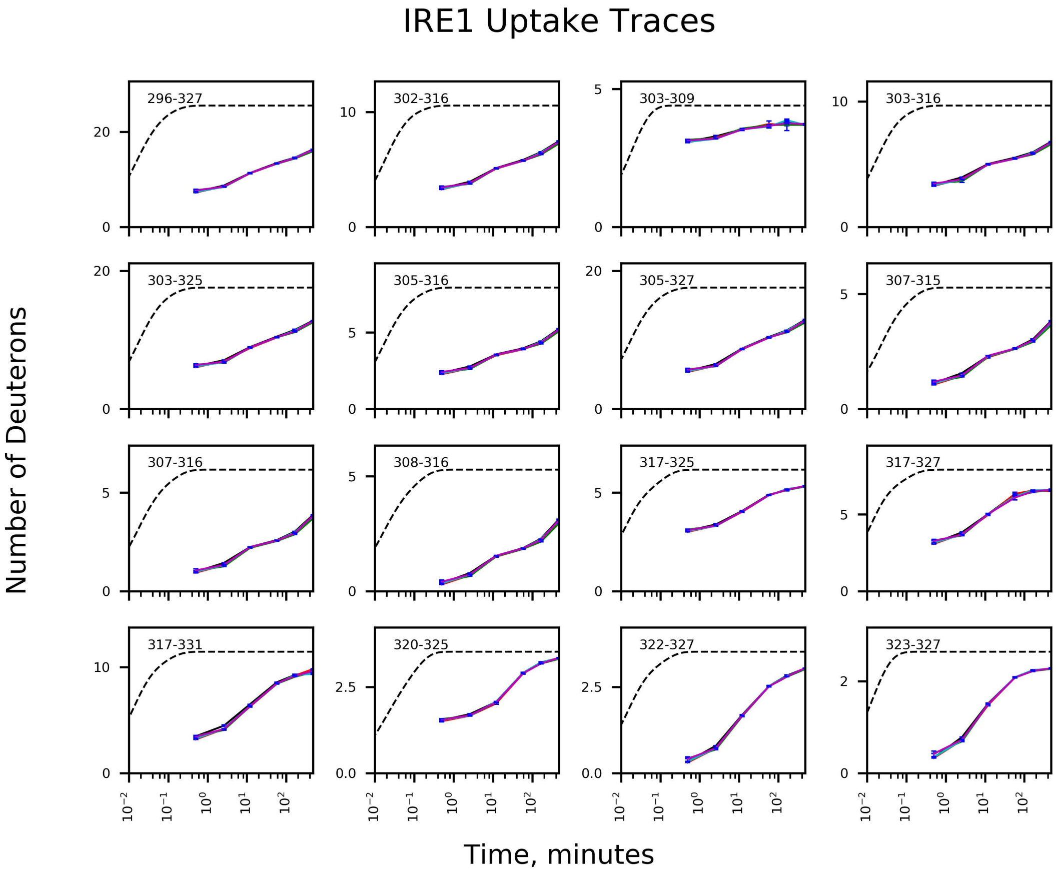

IRE1 Uptake Traces
296-327
302-316
303-309
303-316
303-325
305-316
305-327
307-315
307-316
308-316
317-325
317-327
317-331
320-325
322-327
323-327
Number of Deuterons
Time, minutes

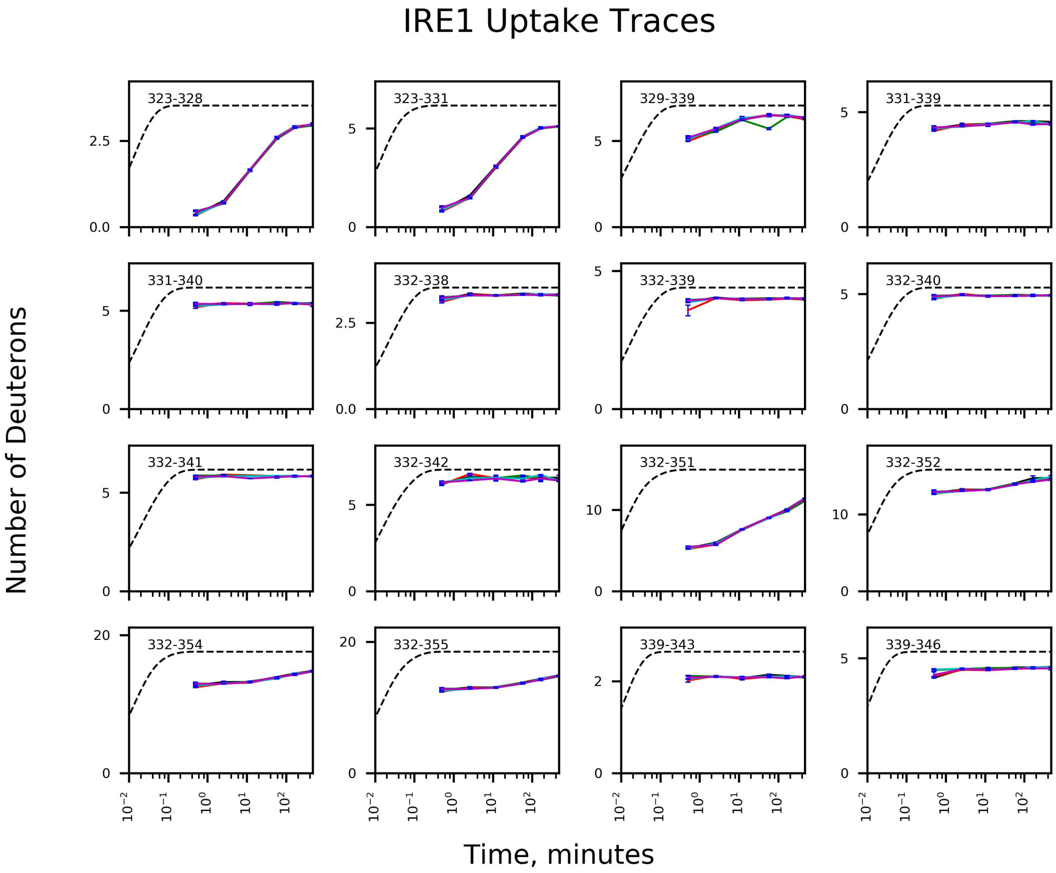

IRE1 Uptake Traces
323-328
323-331
329-339
331-339
331-340
332-338
332-339
332-340
332-341
332-342
332-351
332-352
332-354
332-355
339-343
339-346
Number of Deuterons
Time, minutes

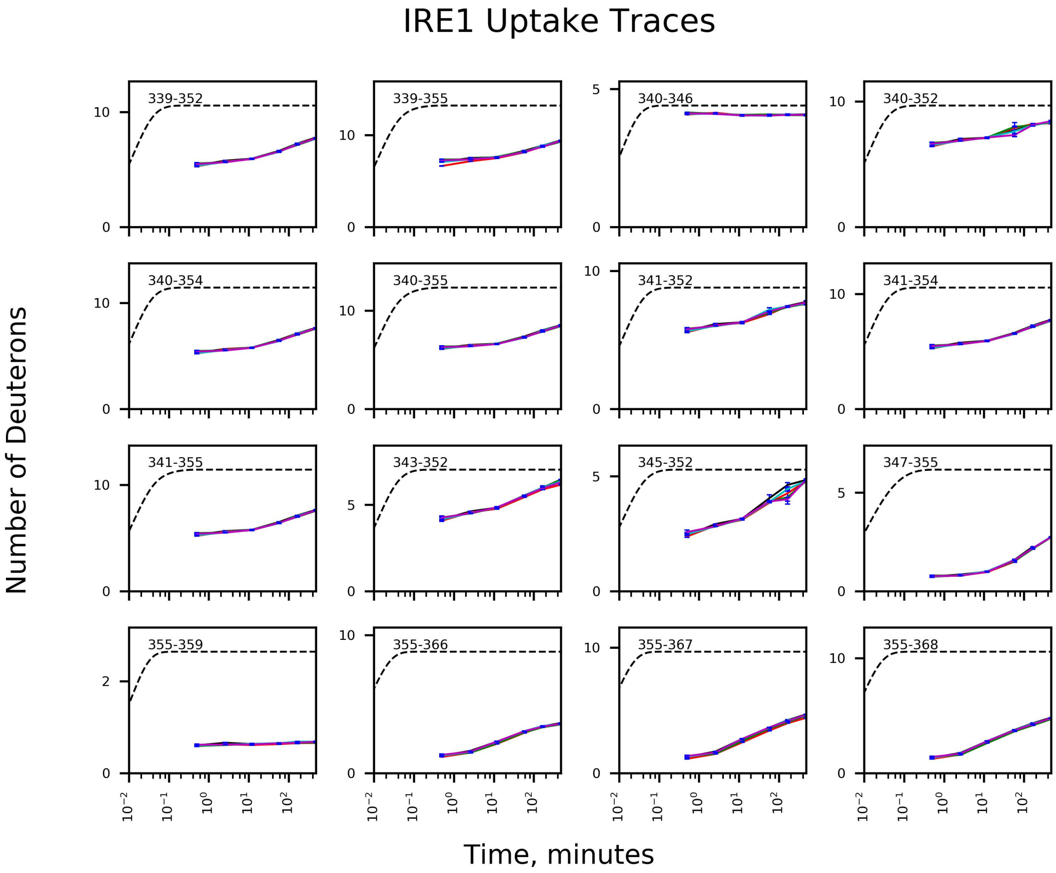

IRE1 Uptake Traces
339-352
339-355
340-346
340-352
340-354
340-355
341-352
341-354
341-355
343-352
345-352
347-355
355-359
355-366
355-367
355-368
Number of Deuterons
Time, minutes

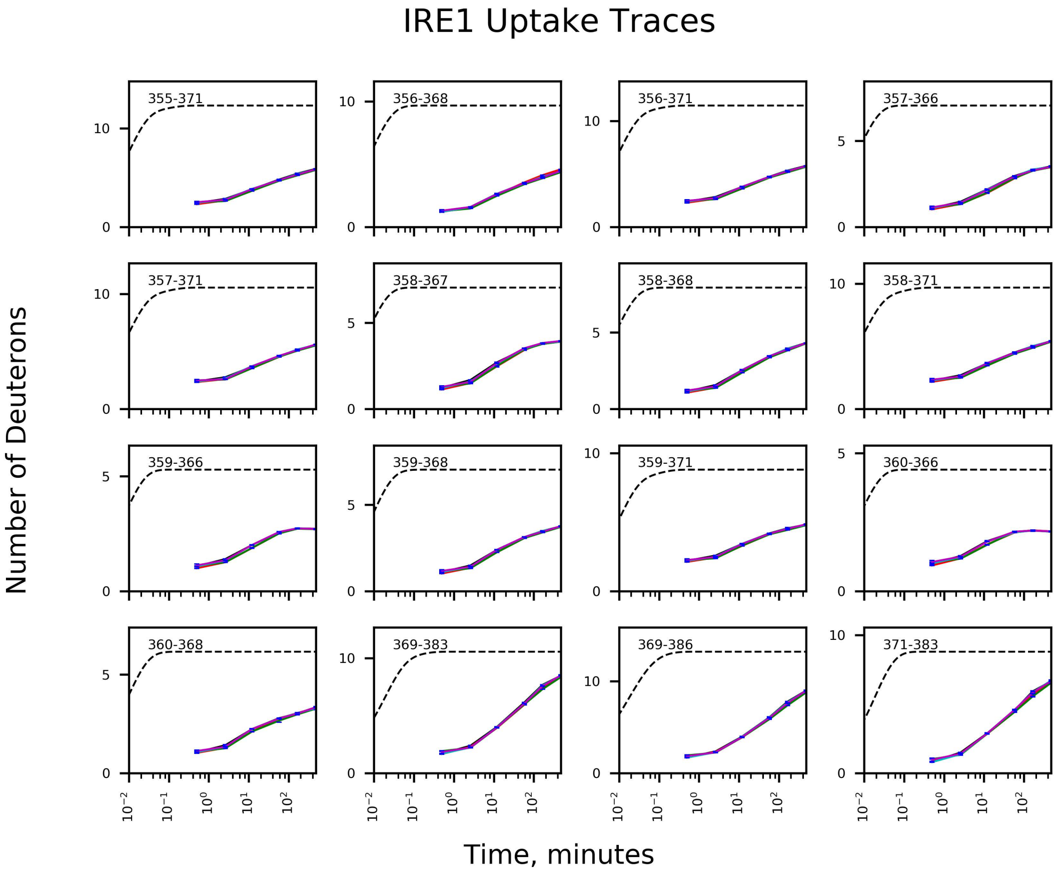
IRE1 Uptake Traces
Number of Deuterons
Time, minutes
355-371
356-368
356-371
357-366
357-371
358-367
358-368
358-371
359-366
359-368
359-371
360-366
360-368
369-383
369-386
371-383

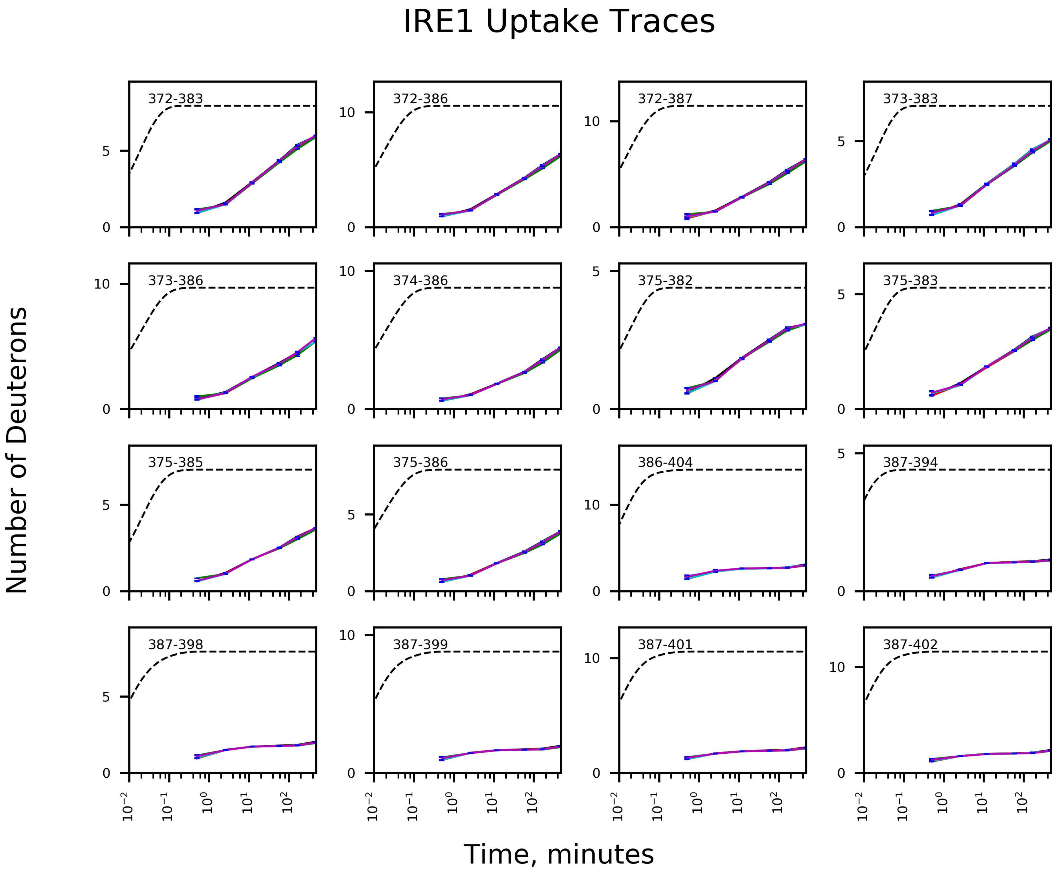

IRE1 Uptake Traces
372-383
372-386
372-387
373-383
373-386
374-386
375-382
375-383
375-385
375-386
386-404
387-394
387-398
387-399
387-401
387-402
Number of Deuterons
Time, minutes

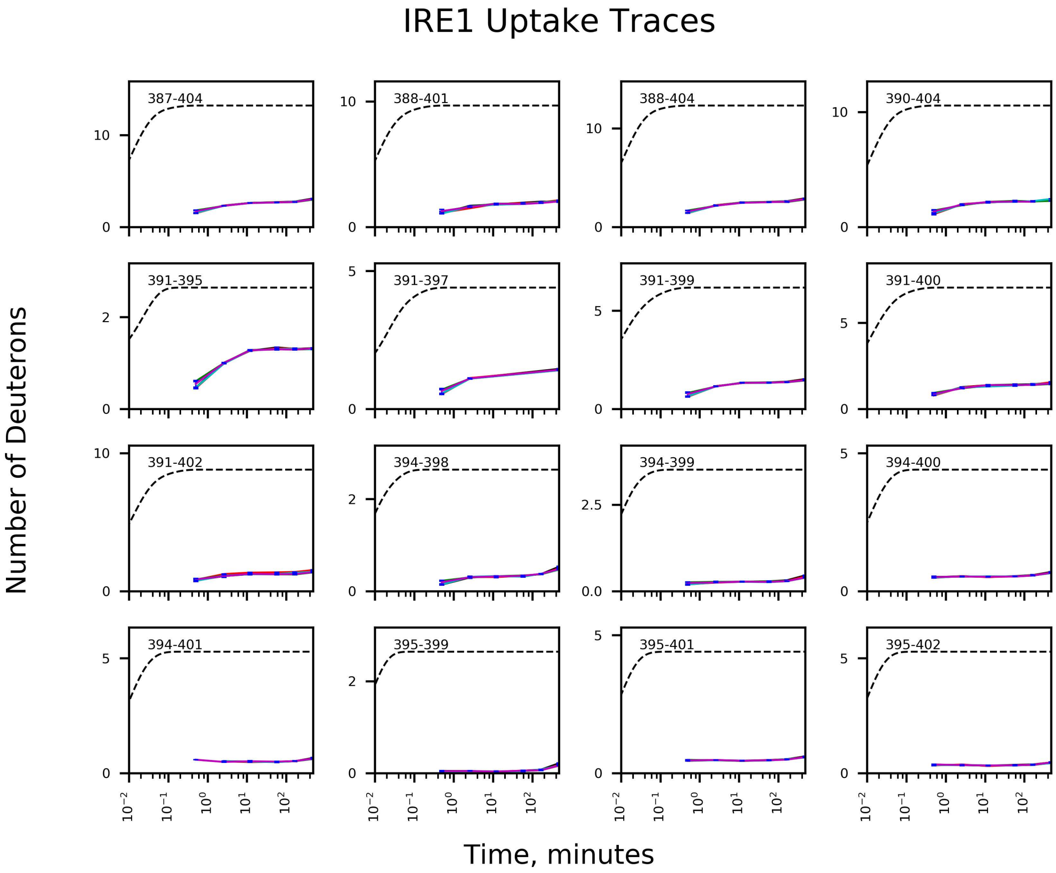
IRE1 Uptake Traces
Number of Deuterons
Time, minutes
387-404
388-401
388-404
390-404
391-395
391-397
391-399
391-400
391-402
394-398
394-399
394-400
394-401
395-399
395-401
395-402

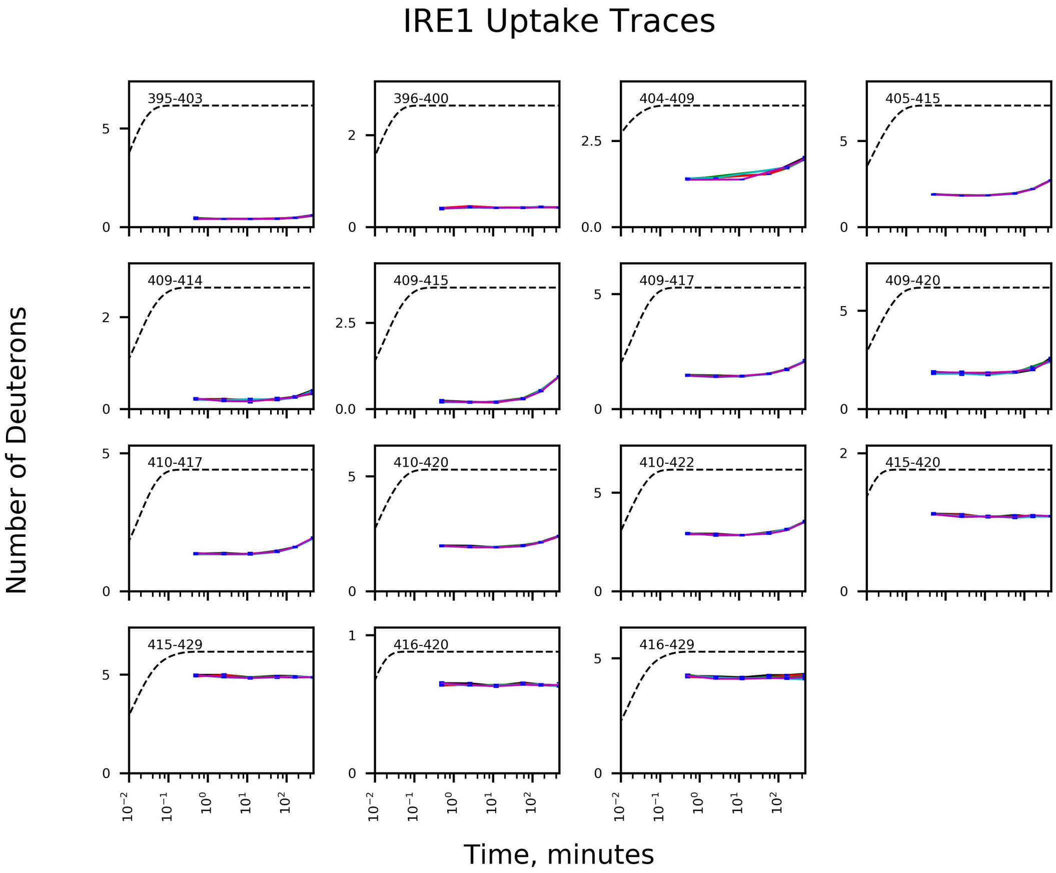

IRE1 Uptake Traces
395-403
396-400
404-409
405-415
409-414
409-415
409-417
409-420
410-417
410-420
410-422
415-420
415-429
416-420
416-429
Number of Deuterons
Time, minutes
