# Supplementary material for: Activation of the IRE1 RNase through remodeling of the kinase front pocket by ATP-competitive ligands
Source: Nat Commun. 2020 Dec 14;11:6387. doi: 10.1038/s41467-020-19974-5 (PMC7736581; doi:10.1038/s41467-020-19974-5)
Supplement: Supplementary file 9 — Reporting Summary [file 41467_2020_19974_MOESM9_ESM.pdf]

nature research

Corresponding author(s): Weiru Wang and Joachim Rudolph

Last updated by author(s): Oct 20, 2020

Reporting Summary

Nature Research wishes to improve the reproducibility of the work that we publish. This form provides structure for consistency and transparency in reporting. For further information on Nature Research policies, seeAuthors & Referees and theEditorial Policy Checklist .

Statistics

For all statistical analyses, confirm that the following items are present in the figure legend, table legend, main text, or Methods section.

n/a	Confirmed	
[ ]	[x]	The exact sample size (*n*) for each experimental group/condition, given as a discrete number and unit of measurement
[ ]	[x]	A statement on whether measurements were taken from distinct samples or whether the same sample was measured repeatedly
[x]	[ ]	The statistical test(s) used AND whether they are one- or two-sided<br>*Only common tests should be described solely by name; describe more complex techniques in the Methods section.*
[x]	[ ]	A description of all covariates tested
[ ]	[x]	A description of any assumptions or corrections, such as tests of normality and adjustment for multiple comparisons
[ ]	[x]	A full description of the statistical parameters including central tendency (e.g. means) or other basic estimates (e.g. regression coefficient) AND variation (e.g. standard deviation) or associated estimates of uncertainty (e.g. confidence intervals)
[x]	[ ]	For null hypothesis testing, the test statistic (e.g. *F*, *t*, *r*) with confidence intervals, effect sizes, degrees of freedom and *P* value noted<br>*Give P values as exact values whenever suitable.*
[x]	[ ]	For Bayesian analysis, information on the choice of priors and Markov chain Monte Carlo settings
[x]	[ ]	For hierarchical and complex designs, identification of the appropriate level for tests and full reporting of outcomes
[x]	[ ]	Estimates of effect sizes (e.g. Cohen's *d*, Pearson's *r*), indicating how they were calculated

Our web collection on statistics for biologists contains articles on many of the points above.

Software and code

Policy information about availability of computer code

Data collection	B3 was used at Advanced Light Source, BLU-Ice was used at Stanford Synchrotron Radiation Lightsource. The software does not have version numbers and it is only available at the indicated beamlines.
Data analysis	iMOSFLM 7.2.2, autoPROC suite 1.1.6/Staraniso 1.10.9, CCP4 7.0, (PHASER 2.8.2, Coot 0.8.9.2), Phenix 1.12, Sedanal 6.67, Prism 7 and 8, PyMol 2.0, Byonic 3.2, Byologic 3.2, MSConvert 3.0.19256-a8cbe7417, ExMS v1.

For manuscripts utilizing custom algorithms or software that are central to the research but not yet described in published literature, software must be made available to editors/reviewers. We strongly encourage code deposition in a community repository (e.g. GitHub). See the Nature Research guidelines for submitting code & software for further information.

Data

Policy information about availability of data

All manuscripts must include a data availability statement. This statement should provide the following information, where applicable:

- Accession codes, unique identifiers, or web links for publicly available datasets
- A list of figures that have associated raw data
- A description of any restrictions on data availability

Coordinates and structure factors of novel structures reported in this manuscript have been deposited in the Protein Data Bank with the following accession codes: 6W39, 6W3A, 6W3B, 6W3C, 6W3E, 6W3K. The source data underlying Figs. 1b-e; 3b,c; 4b,c; 5a,b,e,f; 6 and Supplementary Figs. S1b-d; S5; S6a,b; S7a,b are provided as a Source Data file. The authors declare that all other data supporting the findings of this study are available within the article and its Supplementary Information files, or from the corresponding authors on request.

Field-specific reporting

Please select the one below that is the best fit for your research. If you are not sure, read the appropriate sections before making your selection.

[x] Life sciences   [ ] Behavioural & social sciences   [ ] Ecological, evolutionary & environmental sciences

For a reference copy of the document with all sections, see nature.com/documents/nr-reporting-summary-flat.pdf

Life sciences study design

All studies must disclose on these points even when the disclosure is negative.

Sample size	Every experiment was repeated a minimum of two separate times.
Data exclusions	No data were excluded.
Replication	Samples were replicated in duplicates or triplicates in a single experiment as stated in the methods. Every experiment was repeated a minimum of two separate times.
Randomization	Only biochemical or cell-based experiments (no animals) were carried out in this study and as such there was no need for randomization of our samples.
Blinding	Only biochemical or cell-based experiments (no animals) were carried out in this study and as such there was no need for blinding of our samples.

Reporting for specific materials, systems and methods

We require information from authors about some types of materials, experimental systems and methods used in many studies. Here, indicate whether each material, system or method listed is relevant to your study. If you are not sure if a list item applies to your research, read the appropriate section before selecting a response.

Materials & experimental systems

n/a	Involved in the study	
[ ]	[x]	Antibodies
[ ]	[x]	Eukaryotic cell lines
[x]	[ ]	Palaeontology
[x]	[ ]	Animals and other organisms
[x]	[ ]	Human research participants
[x]	[ ]	Clinical data

Methods

n/a	Involved in the study	
[x]	[ ]	ChIP-seq
[x]	[ ]	Flow cytometry
[x]	[ ]	MRI-based neuroimaging

Antibodies

Antibodies used	Antibody for GAPDH (5174) was from Cell Signaling Technology. Antibodies for detection of IRE1 and XBP1s were generated at Genentech: IRE1α Lumenal Domain (LD) (mouse monoclonal, IgG2a) and XBP1s (rabbit monoclonal): see Shemorry et al. eLife 2019;8:e47084 and Chang et al. j.molcel.2018.06.038 Secondary antibody for western blots (711-035-152, Peroxidase AffiniPure Donkey Anti-Rabbit IgG (H+L)) was from The Jackson Laboratory.
Validation	Antibodies were tested by Western Blots on lysates from various cell lines (including KMS-11 and OPM-2), comparing WT and gene-specific knock-down by RNAi.

Eukaryotic cell lines

Policy information about cell lines

Cell line source(s)	KMS-11 and MDA-MB-231 cells were obtained from ATCC and then maintained in an internal repository at Genentech.
Authentication	Cell lines were authenticated by short tandem repeat (STR) profile. STR profiles are determined for each line using the Promega PowerPlex 16 System. This is performed once and compared to external STR profiles of cell lines (when available) to determine cell line ancestry. Loci analyzed: Detection of sixteen loci (fifteen STR loci and Amelogenin for gender identification), including D3S1358, TH01, D21S11, D18S51, Penta E, D5S818, D13S317, D7S820, D16S539, CSF1PO, Penta D, AMEL, vWA, D8S1179 and TPOX.
Mycoplasma contamination	Cell lines were tested to ensure they were mycoplasma-free within 3 months of use. Two methods are used to avoid false positive/negative results: Lonza Mycoalert and Stratagene Mycosensor. Cell growth rates and morphology are also monitored

	for any batch-to-batch changes.
Commonly misidentified lines<br>(See ICLAC register)	None
